# Supplementary material for: Overcoming the widespread flaws in the annotation of vertebrate selenoprotein genes in public databases
Source: PLoS Comput Biol. 2026 Jan 12;22(1):e1013885. doi: 10.1371/journal.pcbi.1013885 (PMC12822975; doi:10.1371/journal.pcbi.1013885)
Supplement: S2 Table — These are arguments available in the Pyaln function score_similarity. For details, see https://pyaln.readthedocs.io/en/latest/alignment.html#pyaln.Alignment.score_similarity (PDF) [file pcbi.1013885.s005.pdf]

	Parameters	Description
**Gaps**	y	Gaps considered as mismatches
	n	Gaps are not considered
	t	Terminal gaps are ignored
	a	Gaps are considered as any other character
**Metrics**	i	ASI (Average Sequence Identity)
	w	AWSI (Average Weighted Sequence Identity)
**Weights**	m	Maximum frequency of non-gap characters
	i	Information content
	q	Quadratic sum

Supplementary table S2. Score similarity parameters tested for *Selenoprofiles orthology*.

These are arguments available in the Pyaln function score_*similarity*. For details, see

https://pyaln.readthedocs.io/en/latest/alignment.html#pyaln.Alignment.score_similarity
