# Supplementary material for: Overcoming the widespread flaws in the annotation of vertebrate selenoprotein genes in public databases
Source: PLoS Comput Biol. 2026 Jan 12;22(1):e1013885. doi: 10.1371/journal.pcbi.1013885 (PMC12822975; doi:10.1371/journal.pcbi.1013885)
Supplement: S3 Table — See manuscript text for explanation. (PDF) [file pcbi.1013885.s006.pdf]

Families	Gaps	Metric	value	N_seqs	Percentage
GPx	y	ASI	1332	4_seqs	82.4767802
DI	y	ASI	1009	4_seqs	100
TR	y	ASI	746	4_seqs	99.2021277
SelW	y	ASI	480	4_seqs	98.7654321
GPx	y	ASI	1390	8_seqs	86.0681115
DI	y	ASI	1007	8_seqs	99.8017839
TR	y	ASI	746	8_seqs	99.2021277
SelW	y	ASI	481	8_seqs	98.9711934
GPx	y	ASI	1355	12_seqs	83.9009288
DI	y	ASI	1009	12_seqs	100
TR	y	ASI	745	12_seqs	99.0691489
SelW	y	ASI	481	12_seqs	98.9711934
GPx	y	AWSI.i	1274	4_seqs	78.8854489
DI	y	AWSI.i	1009	4_seqs	100
TR	y	AWSI.i	737	4_seqs	98.0053191
SelW	y	AWSI.i	480	4_seqs	98.7654321
GPx	y	AWSI.i	1300	8_seqs	80.495356
DI	y	AWSI.i	999	8_seqs	99.0089197
TR	y	AWSI.i	745	8_seqs	99.0691489
SelW	y	AWSI.i	482	8_seqs	99.1769547
GPx	y	AWSI.i	1304	12_seqs	80.7430341
DI	y	AWSI.i	1009	12_seqs	100
TR	y	AWSI.i	745	12_seqs	99.0691489
SelW	y	AWSI.i	481	12_seqs	98.9711934
GPx	y	AWSI.q	1298	4_seqs	80.371517
DI	y	AWSI.q	1006	4_seqs	99.7026759
TR	y	AWSI.q	744	4_seqs	98.9361702
SelW	y	AWSI.q	480	4_seqs	98.7654321
GPx	y	AWSI.q	1309	8_seqs	81.0526316
DI	y	AWSI.q	1002	8_seqs	99.3062438
TR	y	AWSI.q	746	8_seqs	99.2021277
SelW	y	AWSI.q	481	8_seqs	98.9711934
GPx	y	AWSI.q	1322	12_seqs	81.8575851
DI	y	AWSI.q	1006	12_seqs	99.7026759
TR	y	AWSI.q	744	12_seqs	98.9361702
SelW	y	AWSI.q	441	12_seqs	90.7407407

GPx	y	AWSI.m	1318	4_seqs	81.6099071
DI	y	AWSI.m	1007	4_seqs	99.8017839
TR	y	AWSI.m	745	4_seqs	99.0691489
SelW	y	AWSI.m	439	4_seqs	90.3292181
GPx	y	AWSI.m	1320	8_seqs	81.7337461
DI	y	AWSI.m	1007	8_seqs	99.8017839
TR	y	AWSI.m	745	8_seqs	99.0691489
SelW	y	AWSI.m	441	8_seqs	90.7407407
GPx	y	AWSI.m	1330	12_seqs	82.3529412
DI	y	AWSI.m	1006	12_seqs	99.7026759
TR	y	AWSI.m	745	12_seqs	99.0691489
SelW	y	AWSI.m	440	12_seqs	90.5349794
GPx	n	ASI	1589	4_seqs	98.3900929
DI	n	ASI	1009	4_seqs	100
TR	n	ASI	752	4_seqs	100
SelW	n	ASI	484	4_seqs	99.5884774
GPx	n	ASI	1504	8_seqs	93.126935
DI	n	ASI	1007	8_seqs	99.8017839
TR	n	ASI	752	8_seqs	100
SelW	n	ASI	484	8_seqs	99.5884774
GPx	n	ASI	1591	12_seqs	98.5139319
DI	n	ASI	1009	12_seqs	100
TR	n	ASI	752	12_seqs	100
SelW	n	ASI	485	12_seqs	99.7942387
GPx	n	AWSI.i	1531	4_seqs	94.7987616
DI	n	AWSI.i	1009	4_seqs	100
TR	n	AWSI.i	751	4_seqs	99.8670213
SelW	n	AWSI.i	476	4_seqs	97.9423868
GPx	n	AWSI.i	1403	8_seqs	86.873065
DI	n	AWSI.i	1008	8_seqs	99.900892
TR	n	AWSI.i	752	8_seqs	100
SelW	n	AWSI.i	480	8_seqs	98.7654321
GPx	n	AWSI.i	1493	12_seqs	92.4458204
DI	n	AWSI.i	1009	12_seqs	100
TR	n	AWSI.i	751	12_seqs	99.8670213
SelW	n	AWSI.i	481	12_seqs	98.9711934
GPx	n	AWSI.q	1530	4_seqs	94.7368421
DI	n	AWSI.q	1009	4_seqs	100
TR	n	AWSI.q	752	4_seqs	100

SelW	n	AWSI.q	485	4_seqs	99.7942387
GPx	n	AWSI.q	1534	8_seqs	94.9845201
DI	n	AWSI.q	992	8_seqs	98.3151635
TR	n	AWSI.q	752	8_seqs	100
SelW	n	AWSI.q	485	8_seqs	99.7942387
GPx	n	AWSI.q	1585	12_seqs	98.1424149
DI	n	AWSI.q	1009	12_seqs	100
TR	n	AWSI.q	752	12_seqs	100
SelW	n	AWSI.q	484	12_seqs	99.5884774
GPx	n	AWSI.m	1536	4_seqs	95.1083591
DI	n	AWSI.m	1009	4_seqs	100
TR	n	AWSI.m	752	4_seqs	100
SelW	n	AWSI.m	484	4_seqs	99.5884774
GPx	n	AWSI.m	1557	8_seqs	96.4086687
DI	n	AWSI.m	1007	8_seqs	99.8017839
TR	n	AWSI.m	752	8_seqs	100
SelW	n	AWSI.m	485	8_seqs	99.7942387
GPx	n	AWSI.m	1598	12_seqs	98.9473684
DI	n	AWSI.m	1009	12_seqs	100
TR	n	AWSI.m	752	12_seqs	100
SelW	n	AWSI.m	484	12_seqs	99.5884774
GPx	t	ASI	1355	4_seqs	83.9009288
DI	t	ASI	1009	4_seqs	100
TR	t	ASI	738	4_seqs	98.1382979
SelW	t	ASI	484	4_seqs	99.5884774
GPx	t	ASI	1389	8_seqs	86.006192
DI	t	ASI	1007	8_seqs	99.8017839
TR	t	ASI	735	8_seqs	97.7393617
SelW	t	ASI	484	8_seqs	99.5884774
GPx	t	ASI	1431	12_seqs	88.6068111
DI	t	ASI	1009	12_seqs	100
TR	t	ASI	751	12_seqs	99.8670213
SelW	t	ASI	485	12_seqs	99.7942387
GPx	t	AWSI.i	1299	4_seqs	80.4334365
DI	t	AWSI.i	1006	4_seqs	99.7026759
TR	t	AWSI.i	735	4_seqs	97.7393617
SelW	t	AWSI.i	484	4_seqs	99.5884774
GPx	t	AWSI.i	1310	8_seqs	81.1145511
DI	t	AWSI.i	1006	8_seqs	99.7026759

TR	t	AWSI.i	735	8_seqs	97.7393617
SelW	t	AWSI.i	484	8_seqs	99.5884774
GPx	t	AWSI.i	1379	12_seqs	85.3869969
DI	t	AWSI.i	1006	12_seqs	99.7026759
TR	t	AWSI.i	750	12_seqs	99.7340426
SelW	t	AWSI.i	485	12_seqs	99.7942387
GPx	t	AWSI.q	1530	4_seqs	94.7368421
DI	t	AWSI.q	1009	4_seqs	100
TR	t	AWSI.q	752	4_seqs	100
SelW	t	AWSI.q	485	4_seqs	99.7942387
GPx	t	AWSI.q	1516	8_seqs	93.869969
DI	t	AWSI.q	1002	8_seqs	99.3062438
TR	t	AWSI.q	752	8_seqs	100
SelW	t	AWSI.q	485	8_seqs	99.7942387
GPx	t	AWSI.q	1585	12_seqs	98.1424149
DI	t	AWSI.q	1009	12_seqs	100
TR	t	AWSI.q	752	12_seqs	100
SelW	t	AWSI.q	481	12_seqs	98.9711934
GPx	t	AWSI.m	1513	4_seqs	93.6842105
DI	t	AWSI.m	1009	4_seqs	100
TR	t	AWSI.m	752	4_seqs	100
SelW	t	AWSI.m	454	4_seqs	93.4156379
GPx	t	AWSI.m	1401	8_seqs	86.749226
DI	t	AWSI.m	1005	8_seqs	99.6035679
TR	t	AWSI.m	752	8_seqs	100
SelW	t	AWSI.m	481	8_seqs	98.9711934
GPx	t	AWSI.m	1592	12_seqs	98.5758514
DI	t	AWSI.m	1009	12_seqs	100
TR	t	AWSI.m	752	12_seqs	100
SelW	t	AWSI.m	481	12_seqs	98.9711934

Supplementary table S3. Results of benchmark for *Selenoprofiles orthology*. See manuscript text for explanation.
