# Supplementary material for: Overcoming the widespread flaws in the annotation of vertebrate selenoprotein genes in public databases
Source: PLoS Comput Biol. 2026 Jan 12;22(1):e1013885. doi: 10.1371/journal.pcbi.1013885 (PMC12822975; doi:10.1371/journal.pcbi.1013885)
Supplement: S1 Fig — Each row represents a species and each column corresponds to a selenoprotein family. Rectangles represent individual “selenocysteine” Selenoprofiles predictions, color-labeled according to their gene annotation in Ensembl: pink for correctly annotated genes, blue for misannotated genes, grey for genes absent from the Ensembl annotation. Two graphical features are used to indicate the departure from the expected selenoproteome described in 2012 by Mariotti et al (coded in the Expectation_table.csv file at https://github.com/marco-mariotti/selenoprotein_profiles v.1.2.0): yellow represent genes expected in that lineage but undetected by Selenoprofiles; and red crosses mark extra genes compared to expectations, therefore filtered by the Selenoprofiles lineage tool. (PDF) [file pcbi.1013885.s001.pdf]

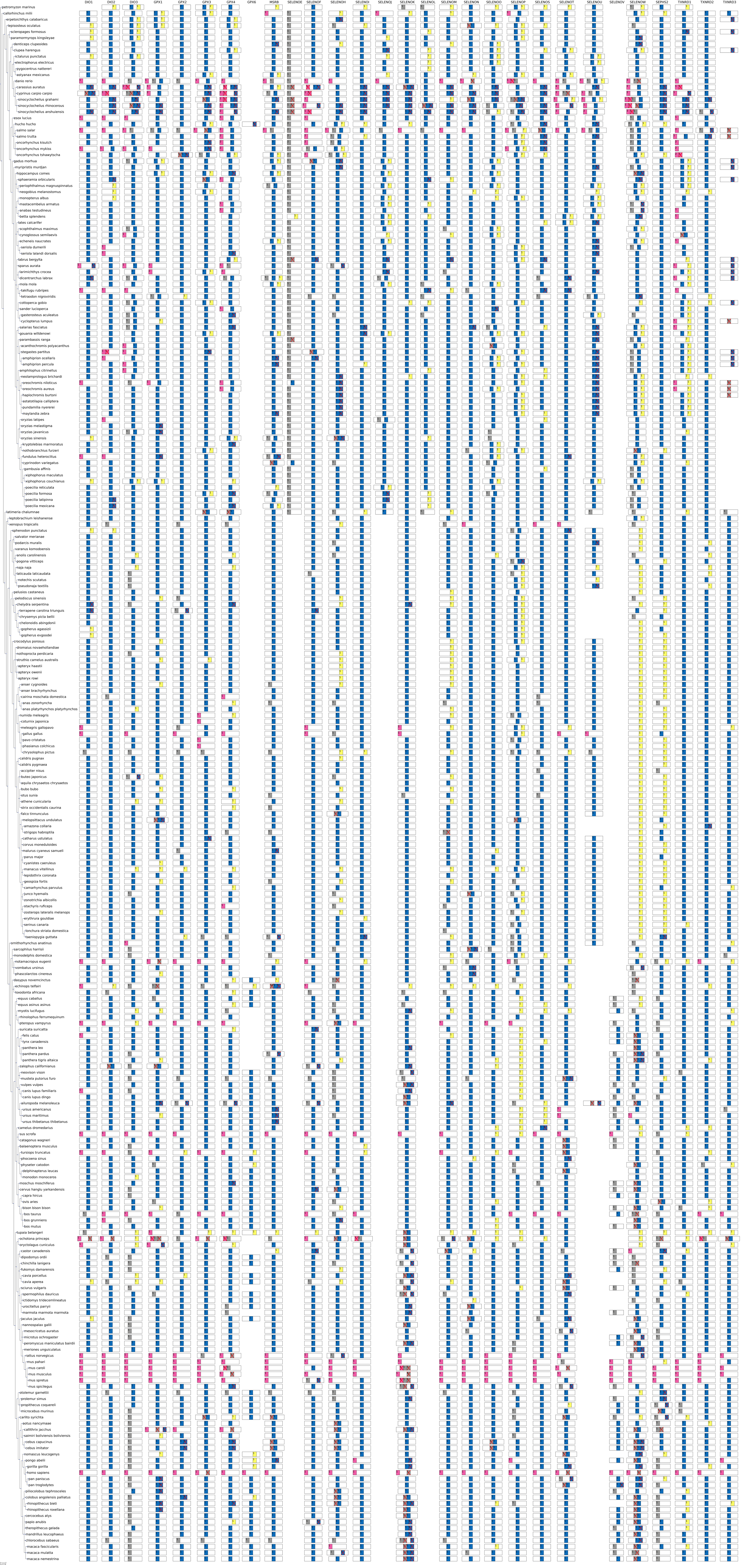
DIO1
DIO2
DIO3
GPX1
GPX2
GPX3
GPX4
GPX6
MSRB
SELENOE
SELENOF
SELENOH
SELENOI
SELENOJ
SELENOK
SELENOL
SELENOM
SELENON
SELENOO
SELENOP
SELENOS
SELENOT
SELENOU
SELENOV
SELENOW
SEPHS2
TXNRD1
TXNRD2
TXNRD3
petromyzon marinus
callorhinchus milii
erpetoichthys calabaricus
lepisosteus oculatus
scleropages formosus
paramormyrops kingsleyae
denticeps clupeoides
clupea harengus
ictalurus punctatus
electrophorus electricus
pygocentrus nattereri
astyanax mexicanus
danio rerio
carassius auratus
cyprinus carpio carpio
sinocyclocheilus grahami
sinocyclocheilus rhinocerous
sinocyclocheilus anshuiensis
esox lucius
hucho hucho
salmo salar
salmo trutta
oncorhynchus kisutch
oncorhynchus mykiss
oncorhynchus tshawytscha
gadus morhua
myripristis murdjan
hippocampus comes
sphaeramia orbicularis
periophthalmus magnuspinnatus
neogobius melanostomus
monopterus albus
mastacembelus armatus
anabas testudineus
betta splendens
lates calcarifer
scophthalmus maximus
cynoglossus semilaevis
echeneis naucrates
seriola dumerili
seriola lalandi dorsalis
labrus bergylta
sparus aurata
larimichthys crocea
dicentrarchus labrax
mola mola
takifugu rubripes
tetraodon nigroviridis
cottoperca gobio
sander lucioperca
gasterosteus aculeatus
cyclopterus lumpus
salarias fasciatus
gouania willdenowi
parambassis ranga
acanthochromis polyacanthus
stegastes partitus
amphiprion ocellaris
amphiprion percula
amphilophus citrinellus
neolamprologus brichardi
oreochromis niloticus
oreochromis aureus
haplochromis burtoni
astatotilapia calliptera
pundamilia nyererei
maylandia zebra
oryzias latipes
oryzias melastigma
oryzias javanicus
oryzias sinensis
kryptolebias marmoratus
nothobranchius furzeri
fundulus heteroclitus
cyprinodon variegatus
gambusia affinis
xiphophorus maculatus
xiphophorus couchianus
poecilia reticulata
poecilia formosa
poecilia latipinna
poecilia mexicana
latimeria chalumnae
leptobrachium leishanense
xenopus tropicalis
sphenodon punctatus
salvator merianae
podarcis muralis
varanus komodoensis
anolis carolinensis
pogona vitticeps
naja naja
laticauda laticaudata
notechis scutatus
pseudonaja textilis
pelusios castaneus
pelodiscus sinensis
chelydra serpentina
terrapene carolina triunguis
chrysemys picta bellii
chelonoidis abingdonii
gopherus agassizii
gopherus evgoodei
crocodylus porosus
dromaius novaehollandiae
nothoprocta perdicaria
struthio camelus australis
apteryx haastii
apteryx owenii
apteryx rowi
anser cygnoides
anser brachyrhynchus
cairina moschata domestica
anas zonorhyncha
anas platyrhynchos platyrhynchos
numida meleagris
coturnix japonica
meleagris gallopavo
gallus gallus
pavo cristatus
phasianus colchicus
chrysolophus pictus
calidris pugnax
calidris pygmaea
accipiter nisus
buteo japonicus
aquila chrysaetos chrysaetos
bubo bubo
otus sunia
athene cunicularia
strix occidentalis caurina
falco tinnunculus
melopsittacus undulatus
amazona collaria
strigops habroptila
catharus ustulatus
corvus moneduloides
malurus cyaneus samueli
parus major
cyanistes caeruleus
manacus vitellinus
lepidothrix coronata
geospiza fortis
camarhynchus parvulus
junco hyemalis
zonotrichia albicollis
stachyris ruficeps
zosterops lateralis melanops
erythrura gouldiae
serinus canaria
lonchura striata domestica
taeniopygia guttata
ornithorhynchus anatinus
sarcophilus harrisii
monodelphis domestica
notamacropus eugenii
vombatus ursinus
phascolarctos cinereus
dasypus novemcinctus
echinops telfairi
loxodonta africana
equus caballus
equus asinus asinus
myotis lucifugus
rhinolophus ferrumequinum
pteropus vampyrus
suricata suricatta
felis catus
lynx canadensis
panthera leo
panthera pardus
panthera tigris altaica
zalophus californianus
neovison vison
mustela putorius furo
vulpes vulpes
canis lupus familiaris
canis lupus dingo
ailuropoda melanoleuca
ursus americanus
ursus maritimus
ursus thibetanus thibetanus
camelus dromedarius
sus scrofa
catagonus wagneri
balaenoptera musculus
tursiops truncatus
phocoena sinus
physeter catodon
delphinapterus leucas
monodon monoceros
moschus moschiferus
cervus hanglu yarkandensis
capra hircus
ovis aries
bison bison bison
bos taurus
bos grunniens
bos mutus
tupaia belangeri
ochotona princeps
oryctolagus cuniculus
castor canadensis
dipodomys ordii
chinchilla lanigera
fukomys damarensis
cavia porcellus
cavia aperea
sciurus vulgaris
spermophilus dauricus
ictidomys tridecemlineatus
urocitellus parryii
marmota marmota marmota
jaculus jaculus
nannospalax galili
mesocricetus auratus
microtus ochrogaster
peromyscus maniculatus bairdii
meriones unguiculatus
rattus norvegicus
mus pahari
mus caroli
mus musculus
mus spretus
mus spicilegus
otolemur garnettii
prolemur simus
propithecus coquereli
microcebus murinus
carlito syrichta
aotus nancymaae
callithrix jacchus
saimiri boliviensis boliviensis
cebus capucinus
cebus imitator
nomascus leucogenys
pongo abelii
gorilla gorilla
homo sapiens
pan paniscus
pan troglodytes
piliocolobus tephrosceles
colobus angolensis palliatus
rhinopithecus bieti
rhinopithecus roxellana
cercocebus atys
papio anubis
theropithecus gelada
mandrillus leucophaeus
chlorocebus sabaeus
macaca fascicularis
macaca mulatta
macaca nemestrina
