# Supplementary material for: Overcoming the widespread flaws in the annotation of vertebrate selenoprotein genes in public databases
Source: PLoS Comput Biol. 2026 Jan 12;22(1):e1013885. doi: 10.1371/journal.pcbi.1013885 (PMC12822975; doi:10.1371/journal.pcbi.1013885)
Supplement: S2 Fig — Each row represents a RefSeq genome assembly and each column corresponds to a selenoprotein family. See caption of S1 Fig for further details. (PDF) [file pcbi.1013885.s002.pdf]

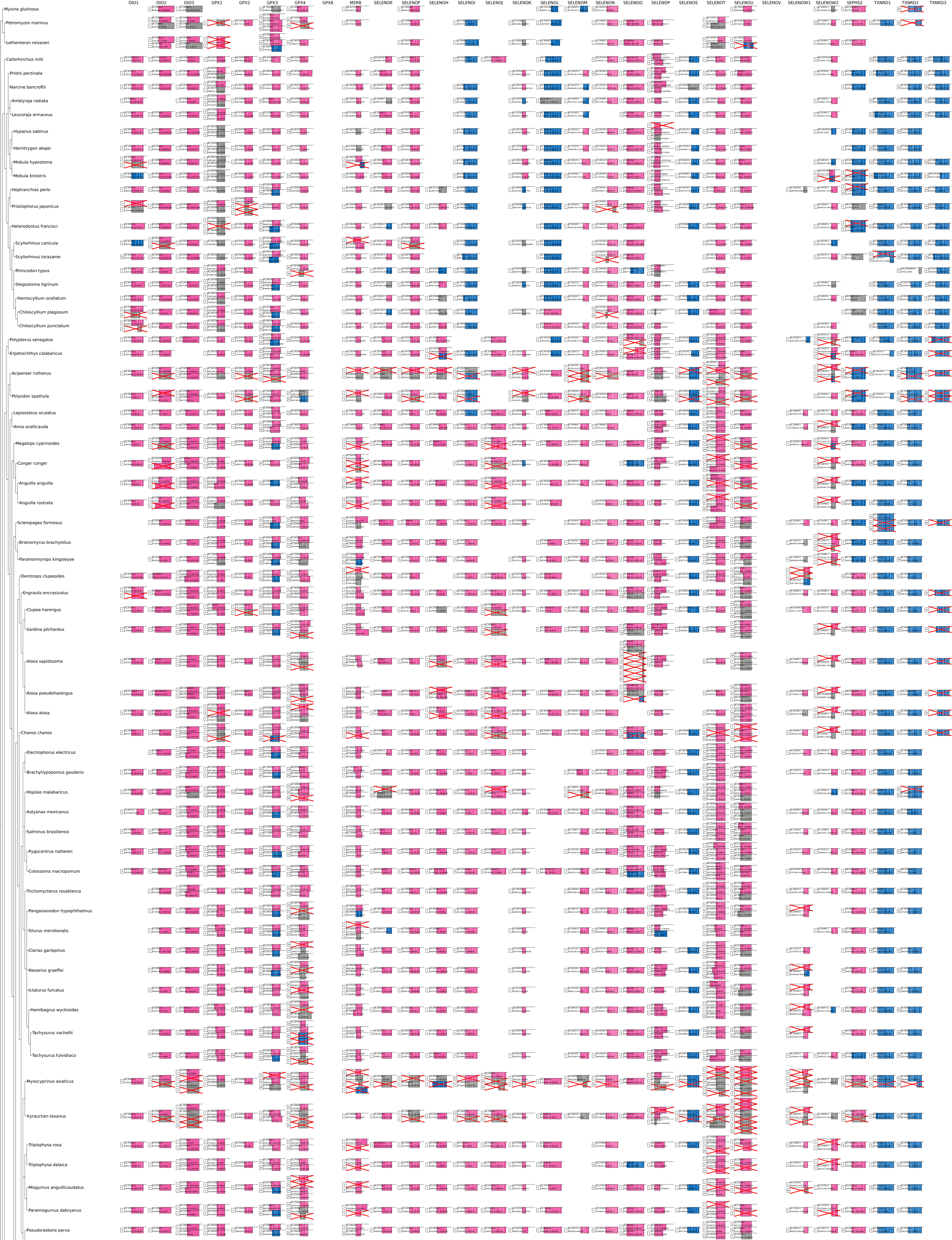
DIO1
DIO2
DIO3
GPX1
GPX2
GPX3
GPX4
GPX6
MSRB
SELENOE
SELENOF
SELENOH
SELENOI
SELENOJ
SELENOK
SELENOL
SELENOM
SELENON
SELENOO
SELENOP
SELENOS
SELENOT
SELENOU
SELENOV
SELENOW1
SELENOW2
SEPHS2
TXNRD1
TXNRD2
TXNRD3
Myxine glutinosa
Petromyzon marinus
Lethenteron reissneri
Callorhinchus milii
Pristis pectinata
Narcine bancroftii
Amblyraja radiata
Leucoraja erinacea
Hypanus sabinus
Hemitrygon akajei
Mobula hypostoma
Mobula birostris
Heptranchias perlo
Pristiophorus japonicus
Heterodontus francisci
Scyliorhinus canicula
Scyliorhinus torazame
Rhincodon typus
Stegostoma tigrinum
Hemiscyllium ocellatum
Chiloscyllium plagiosum
Chiloscyllium punctatum
Polypterus senegalus
Erpetoichthys calabaricus
Acipenser ruthenus
Polyodon spathula
Lepisosteus oculatus
Amia ocellicauda
Megalops cyprinoides
Conger conger
Anguilla anguilla
Anguilla rostrata
Scleropages formosus
Brienomyrus brachyistius
Paramormyrops kingsleyae
Denticeps clupeoides
Engraulis encrasicolus
Clupea harengus
Sardina pilchardus
Alosa sapidissima
Alosa pseudoharengus
Alosa alosa
Chanos chanos
Electrophorus electricus
Brachyhypopomus gauderio
Hoplias malabaricus
Astyanax mexicanus
Salminus brasiliensis
Pygocentrus nattereri
Colossoma macropomum
Trichomycterus rosablanca
Pangasianodon hypophthalmus
Silurus meridionalis
Clarias gariepinus
Neoarius graeffei
Ictalurus furcatus
Hemibagrus wyckioides
Tachysurus vachellii
Tachysurus fulvidraco
Myxocyprinus asiaticus
Xyrauchen texanus
Triplophysa rosa
Triplophysa dalaica
Misgurnus anguillicaudatus
Paramisgurnus dabryanus
Pseudorasbora parva

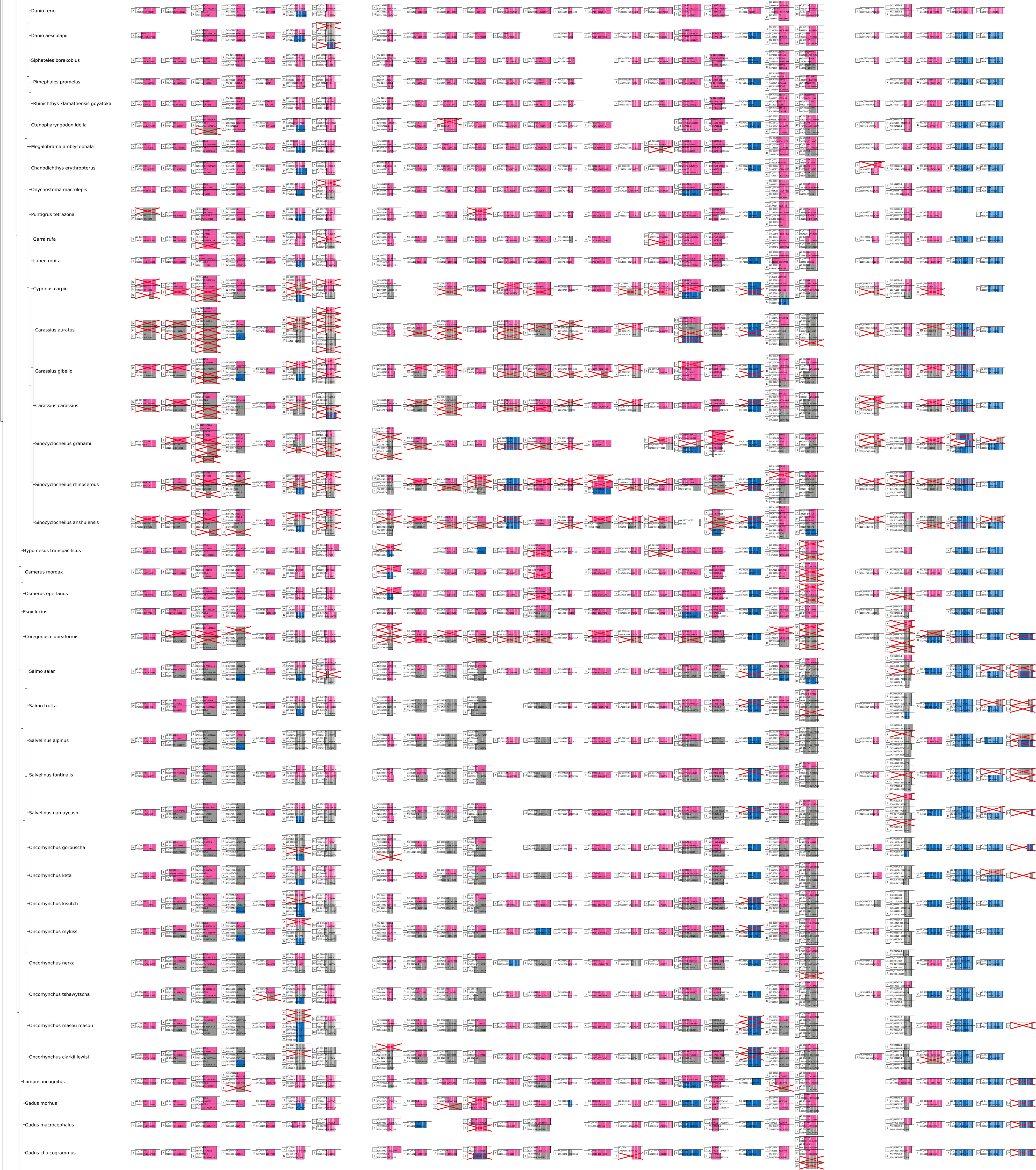
Danio rerio
Danio aesculapii
Siphateles boraxobius
Pimephales promelas
Rhinichthys klamathensis goyatoka
Ctenopharyngodon idella
Megalobrama amblycephala
Chanodichthys erythropterus
Onychostoma macrolepis
Puntigrus tetrazona
Garra rufa
Labeo rohita
Cyprinus carpio
Carassius auratus
Carassius gibelio
Carassius carassius
Sinocyclocheilus grahami
Sinocyclocheilus rhinocerous
Sinocyclocheilus anshuiensis
Hypomesus transpacificus
Osmerus mordax
Osmerus eperlanus
Esox lucius
Coregonus clupeaformis
Salmo salar
Salmo trutta
Salvelinus alpinus
Salvelinus fontinalis
Salvelinus namaycush
Oncorhynchus gorbuscha
Oncorhynchus keta
Oncorhynchus kisutch
Oncorhynchus mykiss
Oncorhynchus nerka
Oncorhynchus tshawytscha
Oncorhynchus masou masou
Oncorhynchus clarkii lewisi
Lampris incognitus
Gadus morhua
Gadus macrocephalus
Gadus chalcogrammus

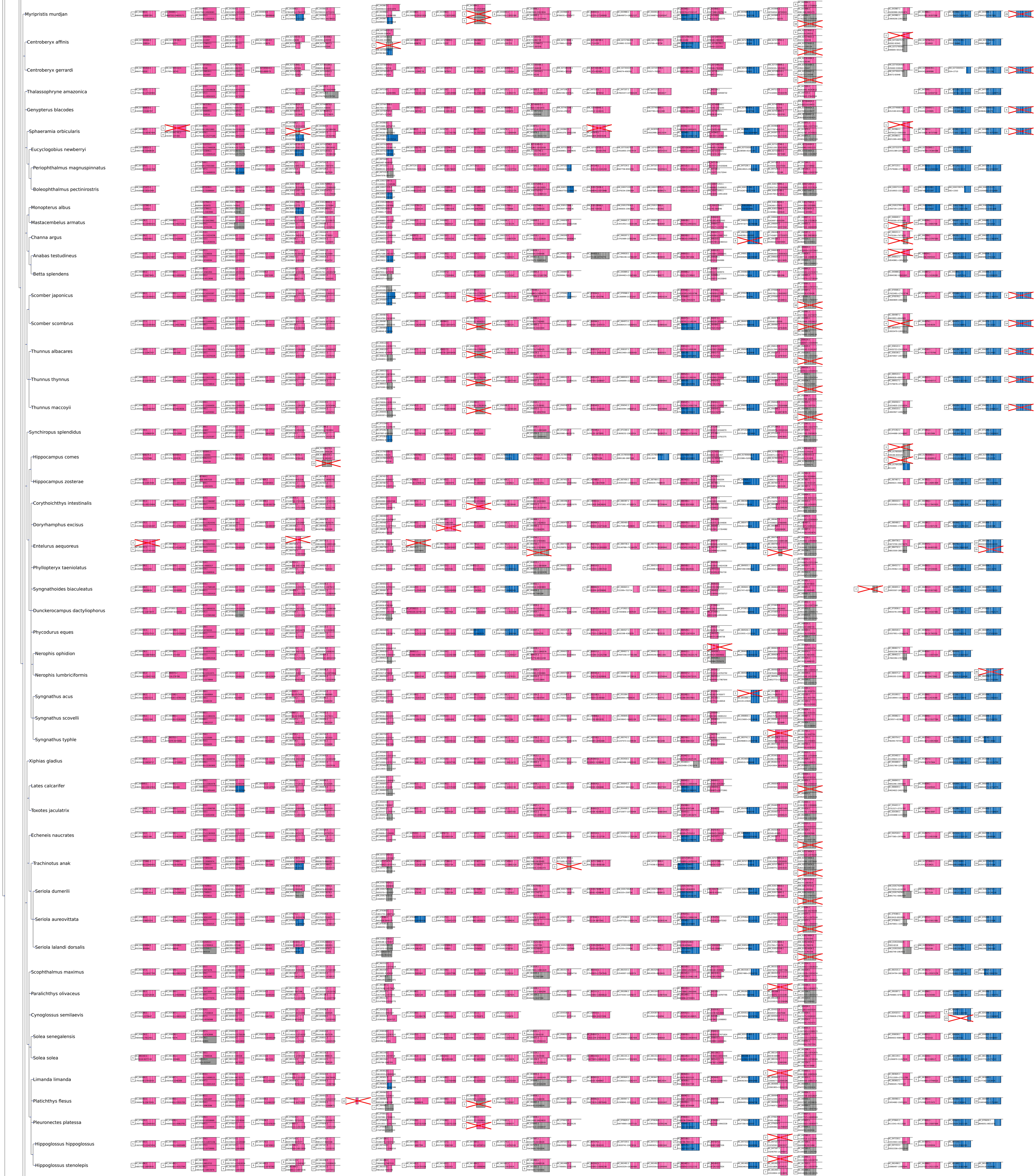
Myripristis murdjan
Centroberyx affinis
Centroberyx gerrardi
Thalassophryne amazonica
Genypterus blacodes
Sphaeramia orbicularis
Eucyclogobius newberryi
Periophthalmus magnuspinnatus
Boleophthalmus pectinirostris
Monopterus albus
Mastacembelus armatus
Channa argus
Anabas testudineus
Betta splendens
Scomber japonicus
Scomber scombrus
Thunnus albacares
Thunnus thynnus
Thunnus maccoyii
Synchiropus splendidus
Hippocampus comes
Hippocampus zosterae
Corythoichthys intestinalis
Doryrhamphus excisus
Entelurus aequoreus
Phyllopteryx taeniolatus
Syngnathoides biaculeatus
Dunckerocampus dactyliophorus
Phycodurus eques
Nerophis ophidion
Nerophis lumbriciformis
Syngnathus acus
Syngnathus scovelli
Syngnathus typhle
Xiphias gladius
Lates calcarifer
Toxotes jaculatrix
Echeneis naucrates
Trachinotus anak
Seriola dumerili
Seriola aureovittata
Seriola lalandi dorsalis
Scophthalmus maximus
Paralichthys olivaceus
Cynoglossus semilaevis
Solea senegalensis
Solea solea
Limanda limanda
Platichthys flesus
Pleuronectes platessa
Hippoglossus hippoglossus
Hippoglossus stenolepis

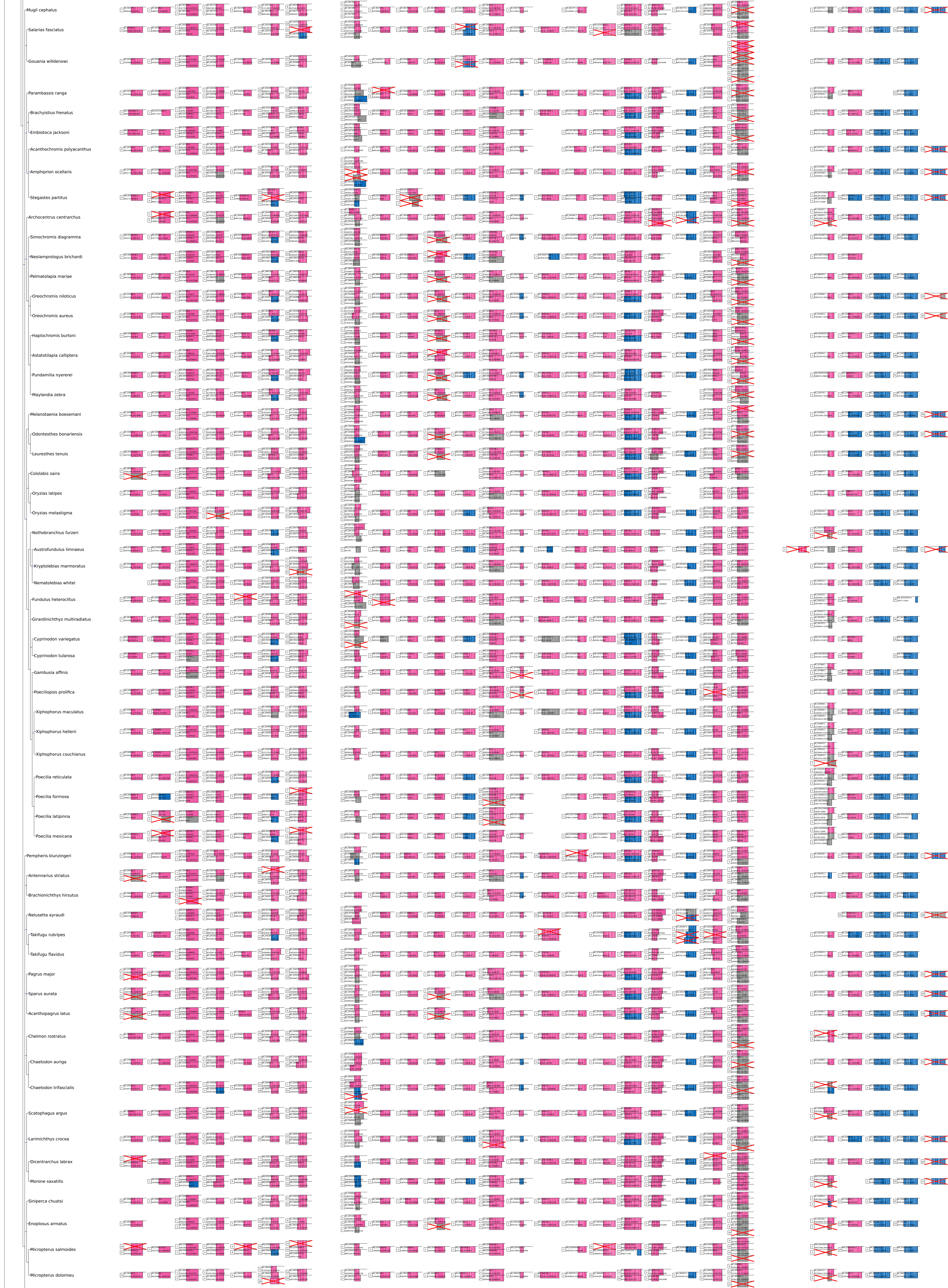
Mugil cephalus
Salarias fasciatus
Gouania willdenowi
Parambassis ranga
Brachyistius frenatus
Embiotoca jacksoni
Acanthochromis polyacanthus
Amphiprion ocellaris
Stegastes partitus
Archocentrus centrarchus
Simochromis diagramma
Neolamprologus brichardi
Pelmatolapia mariae
Oreochromis niloticus
Oreochromis aureus
Haplochromis burtoni
Astatotilapia calliptera
Pundamilia nyererei
Maylandia zebra
Melanotaenia boesemani
Odontesthes bonariensis
Leuresthes tenuis
Cololabis saira
Oryzias latipes
Oryzias melastigma
Nothobranchius furzeri
Austrofundulus limnaeus
Kryptolebias marmoratus
Nematolebias whitei
Fundulus heteroclitus
Girardinichthys multiradiatus
Cyprinodon variegatus
Cyprinodon tularosa
Gambusia affinis
Poeciliopsis prolifica
Xiphophorus maculatus
Xiphophorus hellerii
Xiphophorus couchianus
Poecilia reticulata
Poecilia formosa
Poecilia latipinna
Poecilia mexicana
Pempheris klunzingeri
Antennarius striatus
Brachionichthys hirsutus
Nelusetta ayraudi
Takifugu rubripes
Takifugu flavidus
Pagrus major
Sparus aurata
Acanthopagrus latus
Chelmon rostratus
Chaetodon auriga
Chaetodon trifascialis
Scatophagus argus
Larimichthys crocea
Dicentrarchus labrax
Morone saxatilis
Siniperca chuatsi
Enoplosus armatus
Micropterus salmoides
Micropterus dolomieu

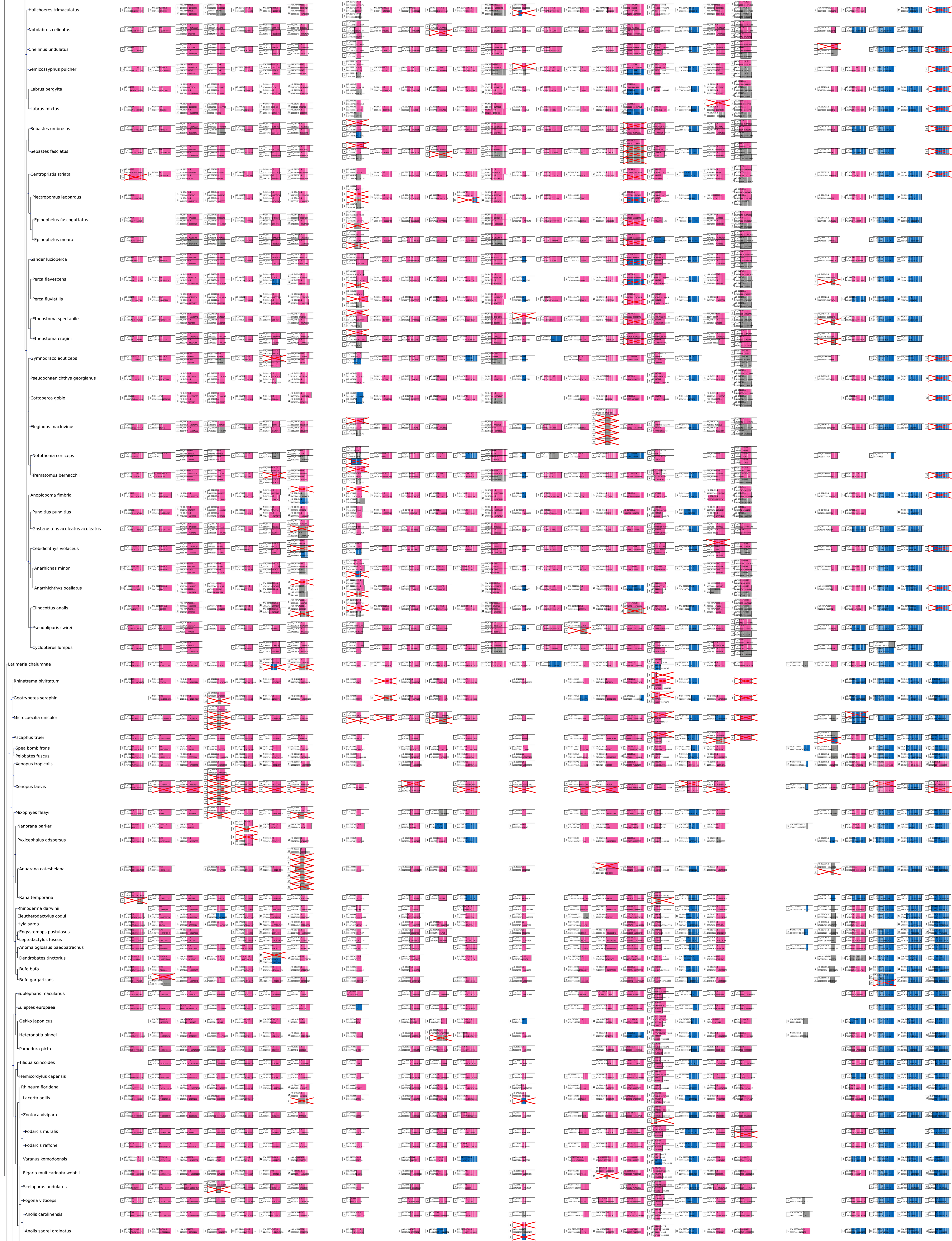

Halichoeres trimaculatus
Notolabrus celidotus
Cheilinus undulatus
Semicossyphus pulcher
Labrus bergylta
Labrus mixtus
Sebastes umbrosus
Sebastes fasciatus
Centropristis striata
Plectropomus leopardus
Epinephelus fuscoguttatus
Epinephelus moara
Sander lucioperca
Perca flavescens
Perca fluviatilis
Etheostoma spectabile
Etheostoma cragini
Gymnodraco acuticeps
Pseudochaenichthys georgianus
Cottoperca gobio
Eleginops maclovinus
Notothenia coriiceps
Trematomus bernacchii
Anoplopoma fimbria
Pungitius pungitius
Gasterosteus aculeatus aculeatus
Cebidichthys violaceus
Anarhichas minor
Anarrhichthys ocellatus
Clinocottus analis
Pseudoliparis swirei
Cyclopterus lumpus
Latimeria chalumnae
Rhinatrema bivittatum
Geotrypetes seraphini
Microcaecilia unicolor
Ascaphus truei
Spea bombifrons
Pelobates fuscus
Xenopus tropicalis
Xenopus laevis
Mixophyes fleayi
Nanorana parkeri
Pyxicephalus adspersus
Aquarana catesbeiana
Rana temporaria
Rhinoderma darwinii
Eleutherodactylus coqui
Hyla sarda
Engystomops pustulosus
Leptodactylus fuscus
Anomaloglossus baeobatrachus
Dendrobates tinctorius
Bufo bufo
Bufo gargarizans
Eublepharis macularius
Euleptes europaea
Gekko japonicus
Heteronotia binoei
Paroedura picta
Tiliqua scincoides
Hemicordylus capensis
Rhineura floridana
Lacerta agilis
Zootoca vivipara
Podarcis muralis
Podarcis raffonei
Varanus komodoensis
Elgaria multicarinata webbii
Sceloporus undulatus
Pogona vitticeps
Anolis carolinensis
Anolis sagrei ordinatus

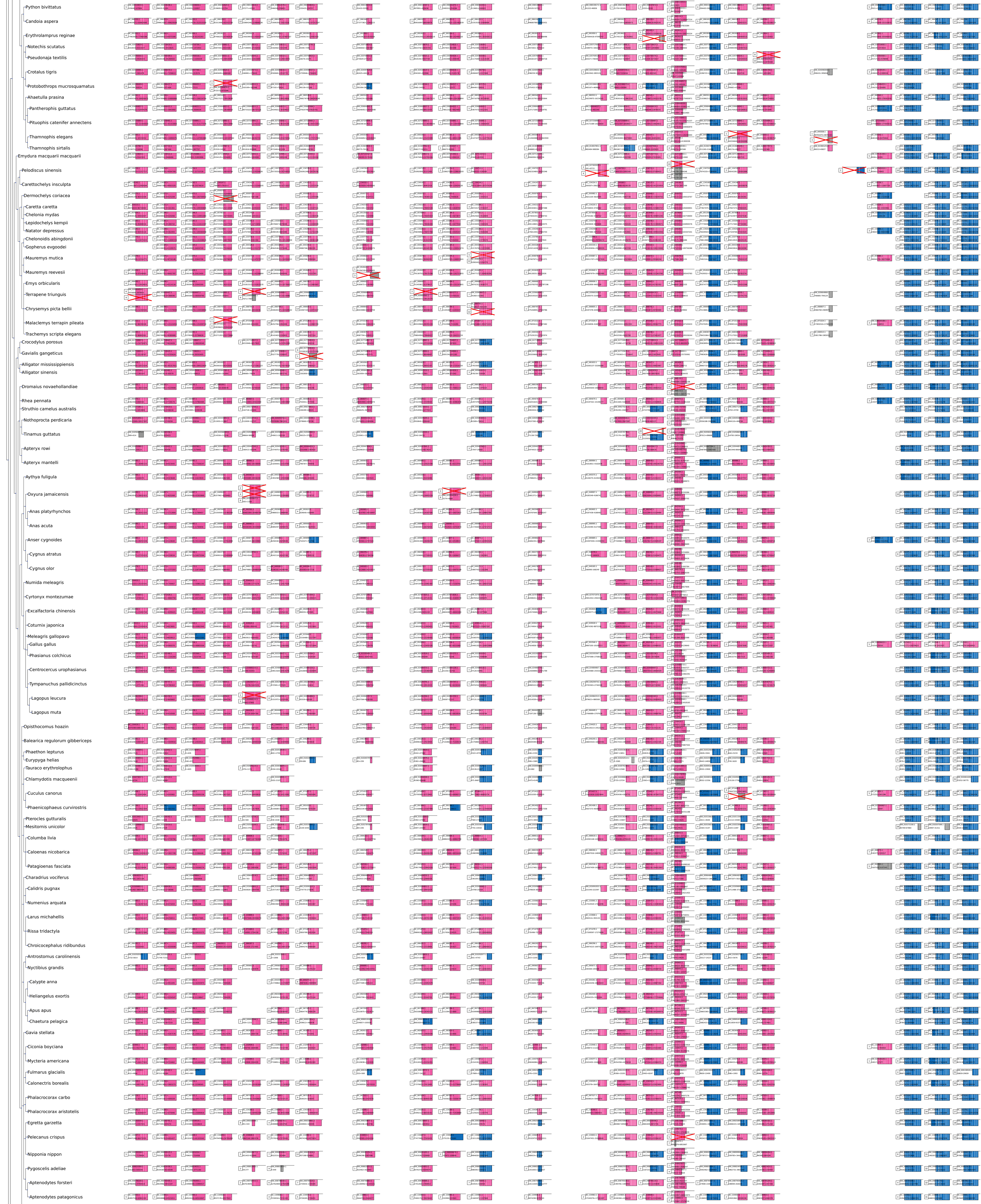
Python bivittatus
Candoia aspera
Erythrolamprus reginae
Notechis scutatus
Pseudonaja textilis
Crotalus tigris
Protobothrops mucrosquamatus
Ahaetulla prasina
Pantherophis guttatus
Pituophis catenifer annectens
Thamnophis elegans
Thamnophis sirtalis
Emydura macquarii macquarii
Pelodiscus sinensis
Carettochelys insculpta
Dermochelys coriacea
Caretta caretta
Chelonia mydas
Lepidochelys kempii
Natator depressus
Chelonoidis abingdonii
Gopherus evgoodei
Mauremys mutica
Mauremys reevesii
Emys orbicularis
Terrapene triunguis
Chrysemys picta bellii
Malaclemys terrapin pileata
Trachemys scripta elegans
Crocodylus porosus
Gavialis gangeticus
Alligator mississippiensis
Alligator sinensis
Dromaius novaehollandiae
Rhea pennata
Struthio camelus australis
Nothoprocta perdicaria
Tinamus guttatus
Apteryx rowi
Apteryx mantelli
Aythya fuligula
Oxyura jamaicensis
Anas platyrhynchos
Anas acuta
Anser cygnoides
Cygnus atratus
Cygnus olor
Numida meleagris
Cyrtonyx montezumae
Excalfactoria chinensis
Coturnix japonica
Meleagris gallopavo
Gallus gallus
Phasianus colchicus
Centrocercus urophasianus
Tympanuchus pallidicinctus
Lagopus leucura
Lagopus muta
Opisthocomus hoazin
Balearica regulorum gibbericeps
Phaethon lepturus
Eurypyga helias
Tauraco erythrolophus
Chlamydotis macqueenii
Cuculus canorus
Phaenicophaeus curvirostris
Pterocles gutturalis
Mesitornis unicolor
Columba livia
Caloenas nicobarica
Patagioenas fasciata
Charadrius vociferus
Calidris pugnax
Numenius arquata
Larus michahellis
Rissa tridactyla
Chroicocephalus ridibundus
Antrostomus carolinensis
Nyctibius grandis
Calypte anna
Heliangelus exortis
Apus apus
Chaetura pelagica
Gavia stellata
Ciconia boyciana
Mycteria americana
Fulmarus glacialis
Calonectris borealis
Phalacrocorax carbo
Phalacrocorax aristotelis
Egretta garzetta
Pelecanus crispus
Nipponia nippon
Pygoscelis adeliae
Aptenodytes forsteri
Aptenodytes patagonicus

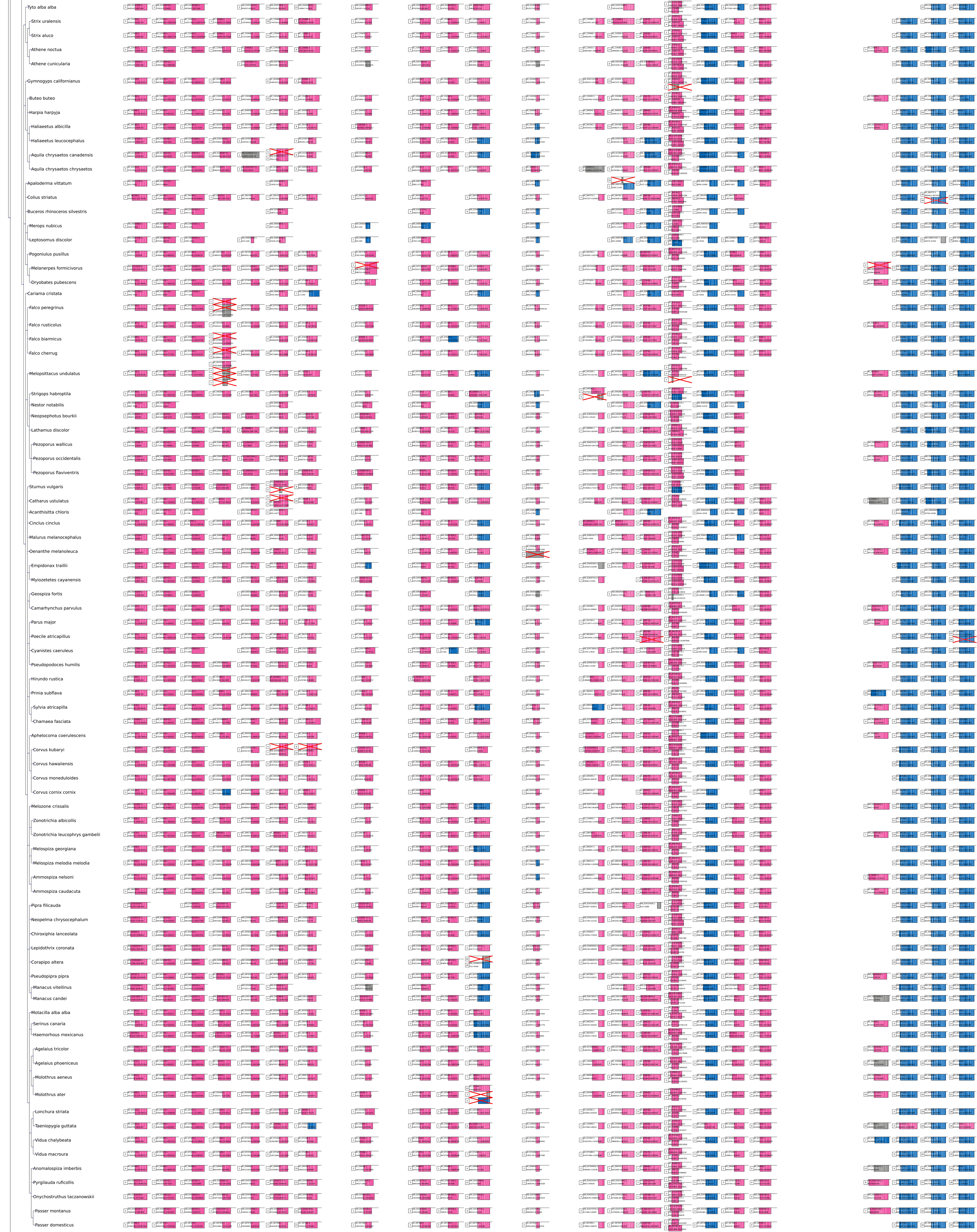

Tyto alba alba
Strix uralensis
Strix aluco
Athene noctua
Athene cunicularia
Gymnogyps californianus
Buteo buteo
Harpia harpyja
Haliaeetus albicilla
Haliaeetus leucocephalus
Aquila chrysaetos canadensis
Aquila chrysaetos chrysaetos
Apaloderma vittatum
Colius striatus
Buceros rhinoceros silvestris
Merops nubicus
Leptosomus discolor
Pogoniulus pusillus
Melanerpes formicivorus
Dryobates pubescens
Cariama cristata
Falco peregrinus
Falco rusticolus
Falco biarmicus
Falco cherrug
Melopsittacus undulatus
Strigops habroptila
Nestor notabilis
Neopsephotus bourkii
Lathamus discolor
Pezoporus wallicus
Pezoporus occidentalis
Pezoporus flaviventris
Sturnus vulgaris
Catharus ustulatus
Acanthisitta chloris
Cinclus cinclus
Malurus melanocephalus
Oenanthe melanoleuca
Empidonax traillii
Myiozetetes cayanensis
Geospiza fortis
Camarhynchus parvulus
Parus major
Poecile atricapillus
Cyanistes caeruleus
Pseudopodoces humilis
Hirundo rustica
Prinia subflava
Sylvia atricapilla
Chamaea fasciata
Aphelocoma coerulescens
Corvus kubaryi
Corvus hawaiiensis
Corvus moneduloides
Corvus cornix cornix
Melozone crissalis
Zonotrichia albicollis
Zonotrichia leucophrys gambelii
Melospiza georgiana
Melospiza melodia melodia
Ammospiza nelsoni
Ammospiza caudacuta
Pipra filicauda
Neopelma chrysocephalum
Chiroxiphia lanceolata
Lepidothrix coronata
Corapipo altera
Pseudopipra pipra
Manacus vitellinus
Manacus candei
Motacilla alba alba
Serinus canaria
Haemorhous mexicanus
Agelaius tricolor
Agelaius phoeniceus
Molothrus aeneus
Molothrus ater
Lonchura striata
Taeniopygia guttata
Vidua chalybeata
Vidua macroura
Anomalospiza imberbis
Pyrgilauda ruficollis
Onychostruthus taczanowskii
Passer montanus
Passer domesticus

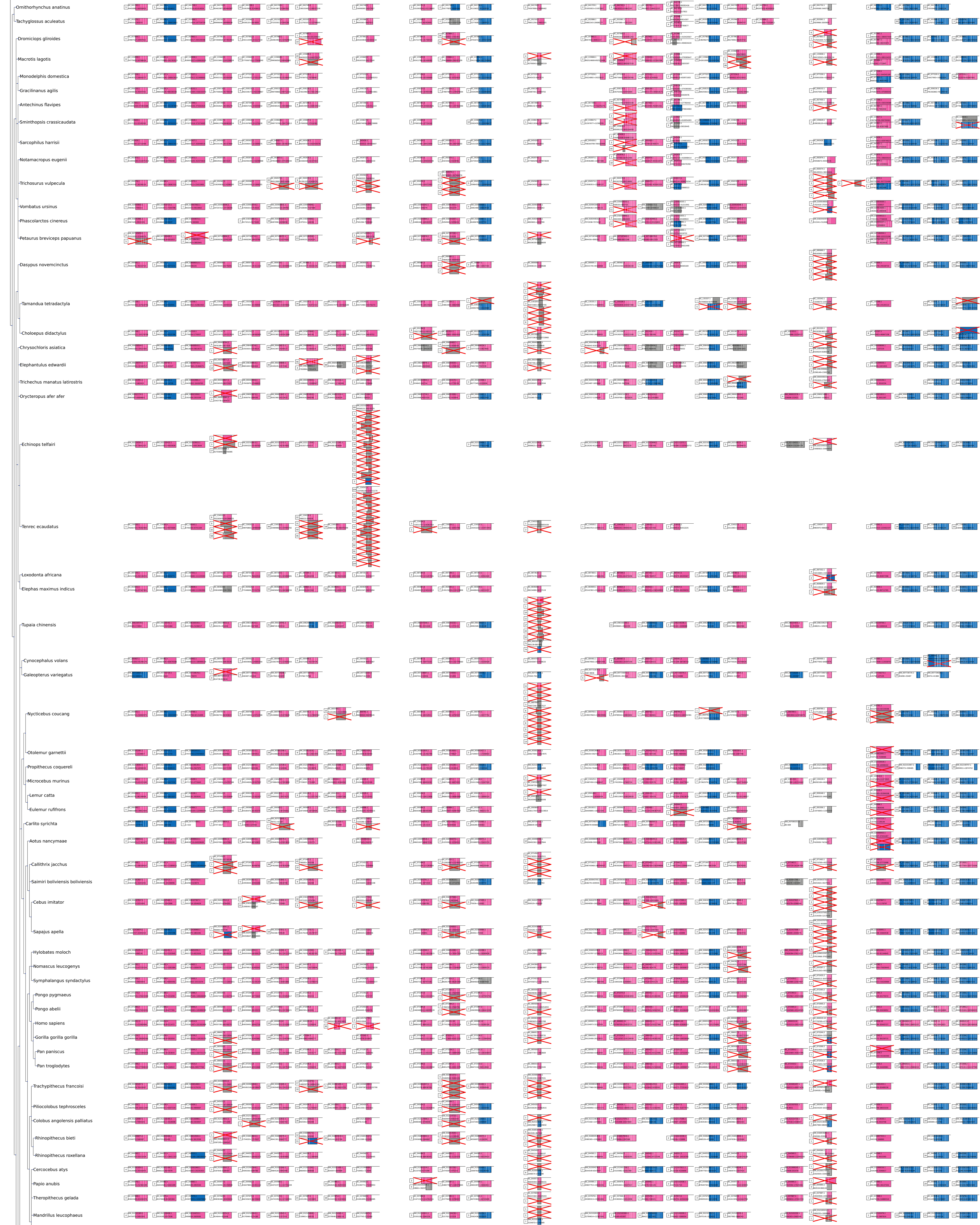

Ornithorhynchus anatinus
Tachyglossus aculeatus
Dromiciops gliroides
Macrotis lagotis
Monodelphis domestica
Gracilinanus agilis
Antechinus flavipes
Sminthopsis crassicaudata
Sarcophilus harrisii
Notamacropus eugenii
Trichosurus vulpecula
Vombatus ursinus
Phascolarctos cinereus
Petaurus breviceps papuanus
Dasypus novemcinctus
Tamandua tetradactyla
Choloepus didactylus
Chrysochloris asiatica
Elephantulus edwardii
Trichechus manatus latirostris
Orycteropus afer afer
Echinops telfairi
Tenrec ecaudatus
Loxodonta africana
Elephas maximus indicus
Tupaia chinensis
Cynocephalus volans
Galeopterus variegatus
Nycticebus coucang
Otolemur garnettii
Propithecus coquereli
Microcebus murinus
Lemur catta
Eulemur rufifrons
Carlito syrichta
Aotus nancymaae
Callithrix jacchus
Saimiri boliviensis boliviensis
Cebus imitator
Sapajus apella
Hylobates moloch
Nomascus leucogenys
Symphalangus syndactylus
Pongo pygmaeus
Pongo abelii
Homo sapiens
Gorilla gorilla gorilla
Pan paniscus
Pan troglodytes
Trachypithecus francoisi
Piliocolobus tephrosceles
Colobus angolensis palliatus
Rhinopithecus bieti
Rhinopithecus roxellana
Cercocebus atys
Papio anubis
Theropithecus gelada
Mandrillus leucophaeus

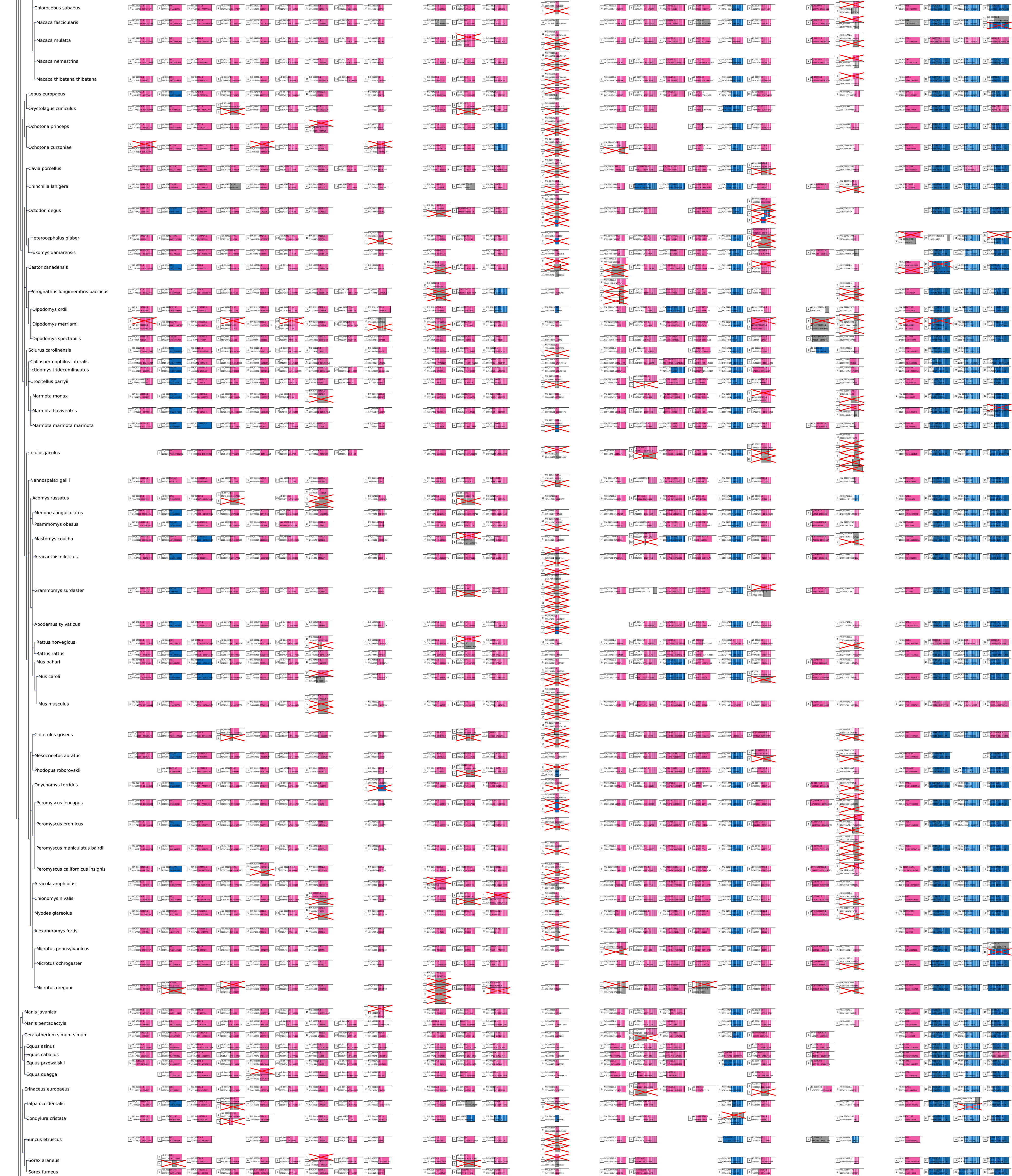
Chlorocebus sabaeus
Macaca fascicularis
Macaca mulatta
Macaca nemestrina
Macaca thibetana thibetana
Lepus europaeus
Oryctolagus cuniculus
Ochotona princeps
Ochotona curzoniae
Cavia porcellus
Chinchilla lanigera
Octodon degus
Heterocephalus glaber
Fukomys damarensis
Castor canadensis
Perognathus longimembris pacificus
Dipodomys ordii
Dipodomys merriami
Dipodomys spectabilis
Sciurus carolinensis
Callospermophilus lateralis
Ictidomys tridecemlineatus
Urocitellus parryii
Marmota monax
Marmota flaviventris
Marmota marmota marmota
Jaculus jaculus
Nannospalax galili
Acomys russatus
Meriones unguiculatus
Psammomys obesus
Mastomys coucha
Arvicanthis niloticus
Grammomys surdaster
Apodemus sylvaticus
Rattus norvegicus
Rattus rattus
Mus pahari
Mus caroli
Mus musculus
Cricetulus griseus
Mesocricetus auratus
Phodopus roborovskii
Onychomys torridus
Peromyscus leucopus
Peromyscus eremicus
Peromyscus maniculatus bairdii
Peromyscus californicus insignis
Arvicola amphibius
Chionomys nivalis
Myodes glareolus
Alexandromys fortis
Microtus pennsylvanicus
Microtus ochrogaster
Microtus oregoni
Manis javanica
Manis pentadactyla
Ceratotherium simum simum
Equus asinus
Equus caballus
Equus przewalskii
Equus quagga
Erinaceus europaeus
Talpa occidentalis
Condylura cristata
Suncus etruscus
Sorex araneus
Sorex fumeus

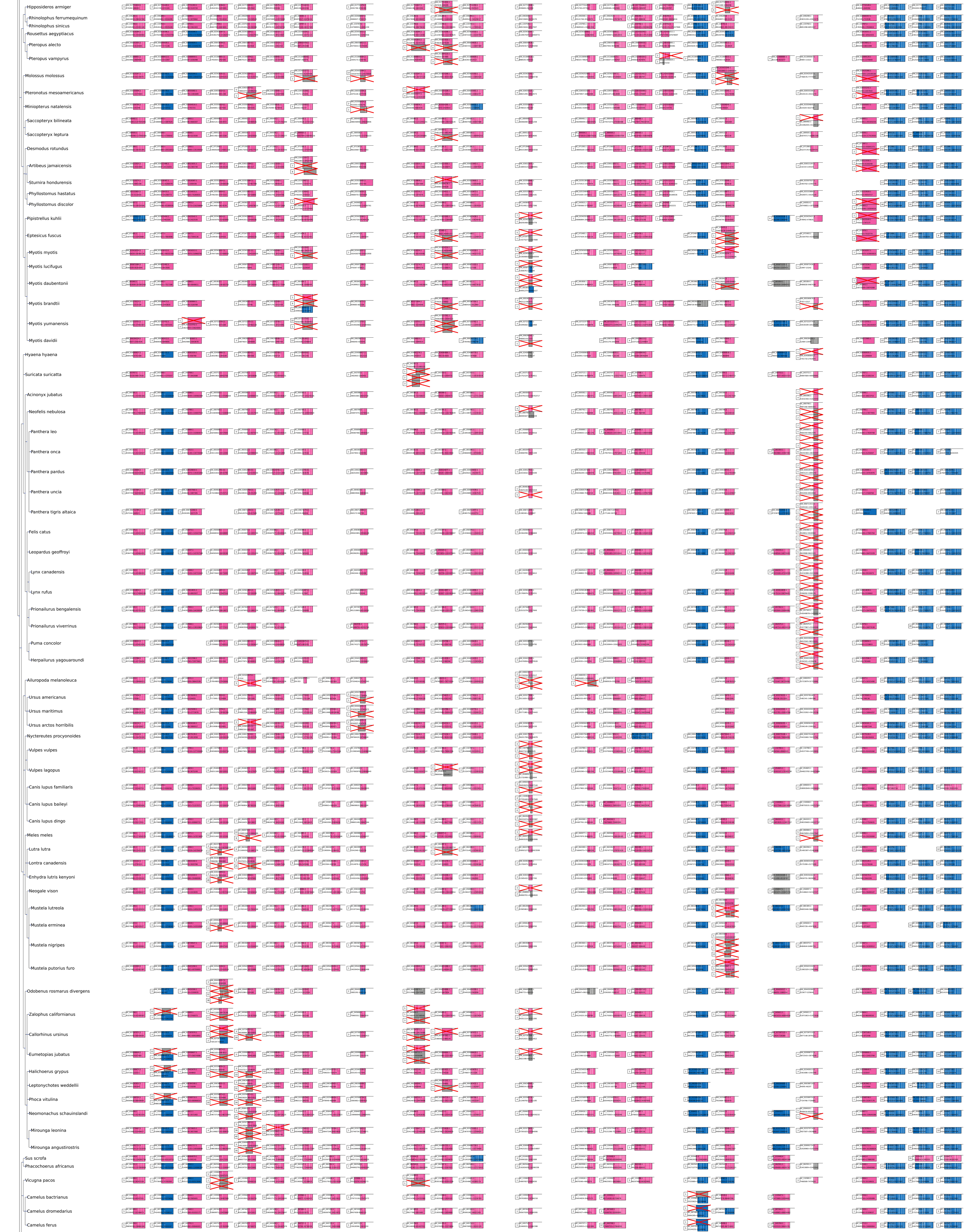
Hipposideros armiger
Rhinolophus ferrumequinum
Rhinolophus sinicus
Rousettus aegyptiacus
Pteropus alecto
Pteropus vampyrus
Molossus molossus
Pteronotus mesoamericanus
Miniopterus natalensis
Saccopteryx bilineata
Saccopteryx leptura
Desmodus rotundus
Artibeus jamaicensis
Sturnira hondurensis
Phyllostomus hastatus
Phyllostomus discolor
Pipistrellus kuhlii
Eptesicus fuscus
Myotis myotis
Myotis lucifugus
Myotis daubentonii
Myotis brandtii
Myotis yumanensis
Myotis davidii
Hyaena hyaena
Suricata suricatta
Acinonyx jubatus
Neofelis nebulosa
Panthera leo
Panthera onca
Panthera pardus
Panthera uncia
Panthera tigris altaica
Felis catus
Leopardus geoffroyi
Lynx canadensis
Lynx rufus
Prionailurus bengalensis
Prionailurus viverrinus
Puma concolor
Herpailurus yagouaroundi
Ailuropoda melanoleuca
Ursus americanus
Ursus maritimus
Ursus arctos horribilis
Nyctereutes procyonoides
Vulpes vulpes
Vulpes lagopus
Canis lupus familiaris
Canis lupus baileyi
Canis lupus dingo
Meles meles
Lutra lutra
Lontra canadensis
Enhydra lutris kenyoni
Neogale vison
Mustela lutreola
Mustela erminea
Mustela nigripes
Mustela putorius furo
Odobenus rosmarus divergens
Zalophus californianus
Callorhinus ursinus
Eumetopias jubatus
Halichoerus grypus
Leptonychotes weddellii
Phoca vitulina
Neomonachus schauinslandi
Mirounga leonina
Mirounga angustirostris
Sus scrofa
Phacochoerus africanus
Vicugna pacos
Camelus bactrianus
Camelus dromedarius
Camelus ferus

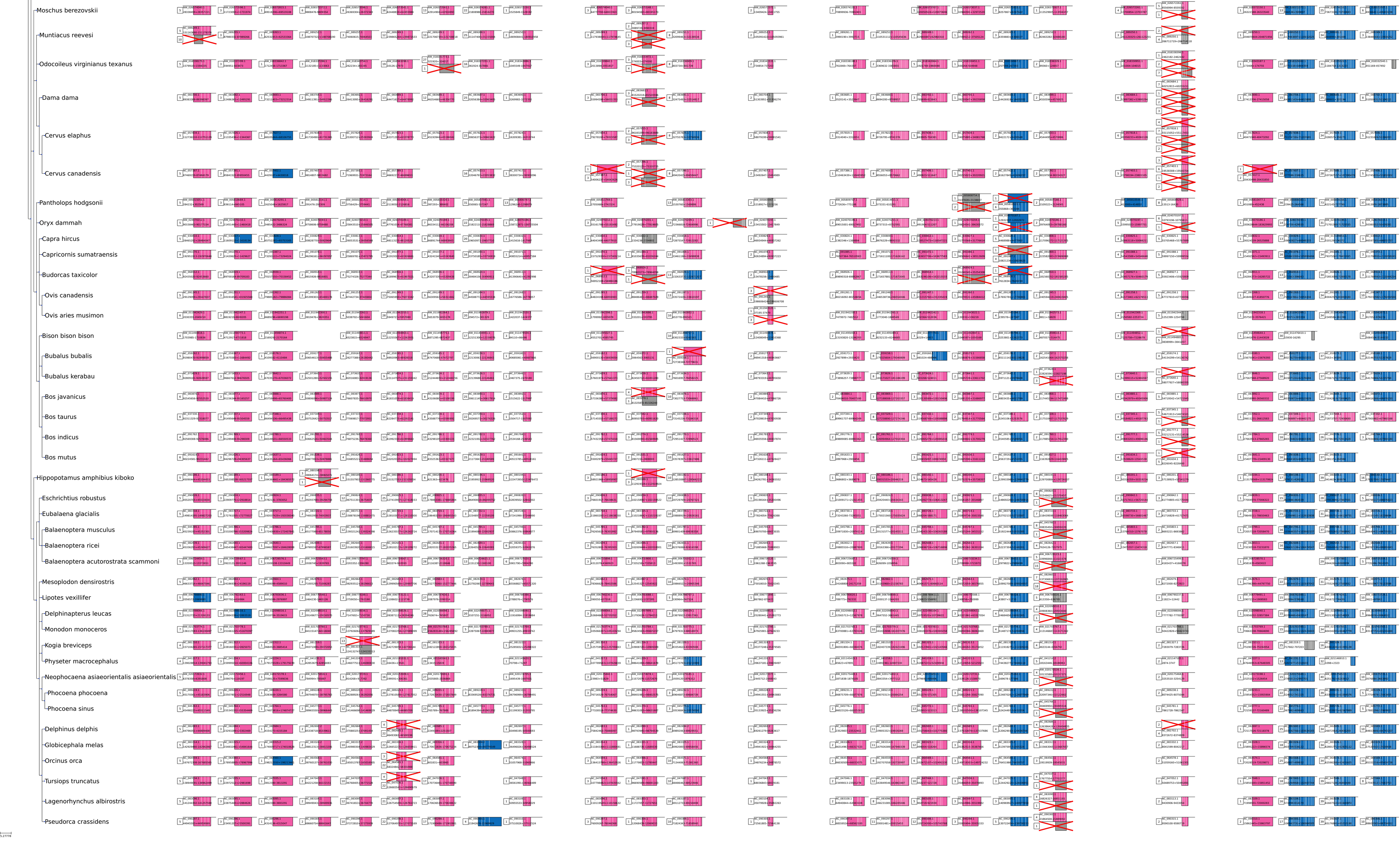
Moschus berezovskii
Muntiacus reevesi
Odocoileus virginianus texanus
Dama dama
Cervus elaphus
Cervus canadensis
Pantholops hodgsonii
Oryx dammah
Capra hircus
Capricornis sumatraensis
Budorcas taxicolor
Ovis canadensis
Ovis aries musimon
Bison bison bison
Bubalus bubalis
Bubalus kerabau
Bos javanicus
Bos taurus
Bos indicus
Bos mutus
Hippopotamus amphibius kiboko
Eschrichtius robustus
Eubalaena glacialis
Balaenoptera musculus
Balaenoptera ricei
Balaenoptera acutorostrata scammoni
Mesoplodon densirostris
Lipotes vexillifer
Delphinapterus leucas
Monodon monoceros
Kogia breviceps
Physeter macrocephalus
Neophocaena asiaeorientalis asiaeorientalis
Phocoena phocoena
Phocoena sinus
Delphinus delphis
Globicephala melas
Orcinus orca
Tursiops truncatus
Lagenorhynchus albirostris
Pseudorca crassidens
