# Supplementary material for: Overcoming the widespread flaws in the annotation of vertebrate selenoprotein genes in public databases
Source: PLoS Comput Biol. 2026 Jan 12;22(1):e1013885. doi: 10.1371/journal.pcbi.1013885 (PMC12822975; doi:10.1371/journal.pcbi.1013885)
Supplement: S3 Fig — Each row represents a GenBank genome assembly and each column corresponds to a selenoprotein family. See caption of S1 Fig for further details. (PDF) [file pcbi.1013885.s003.pdf]

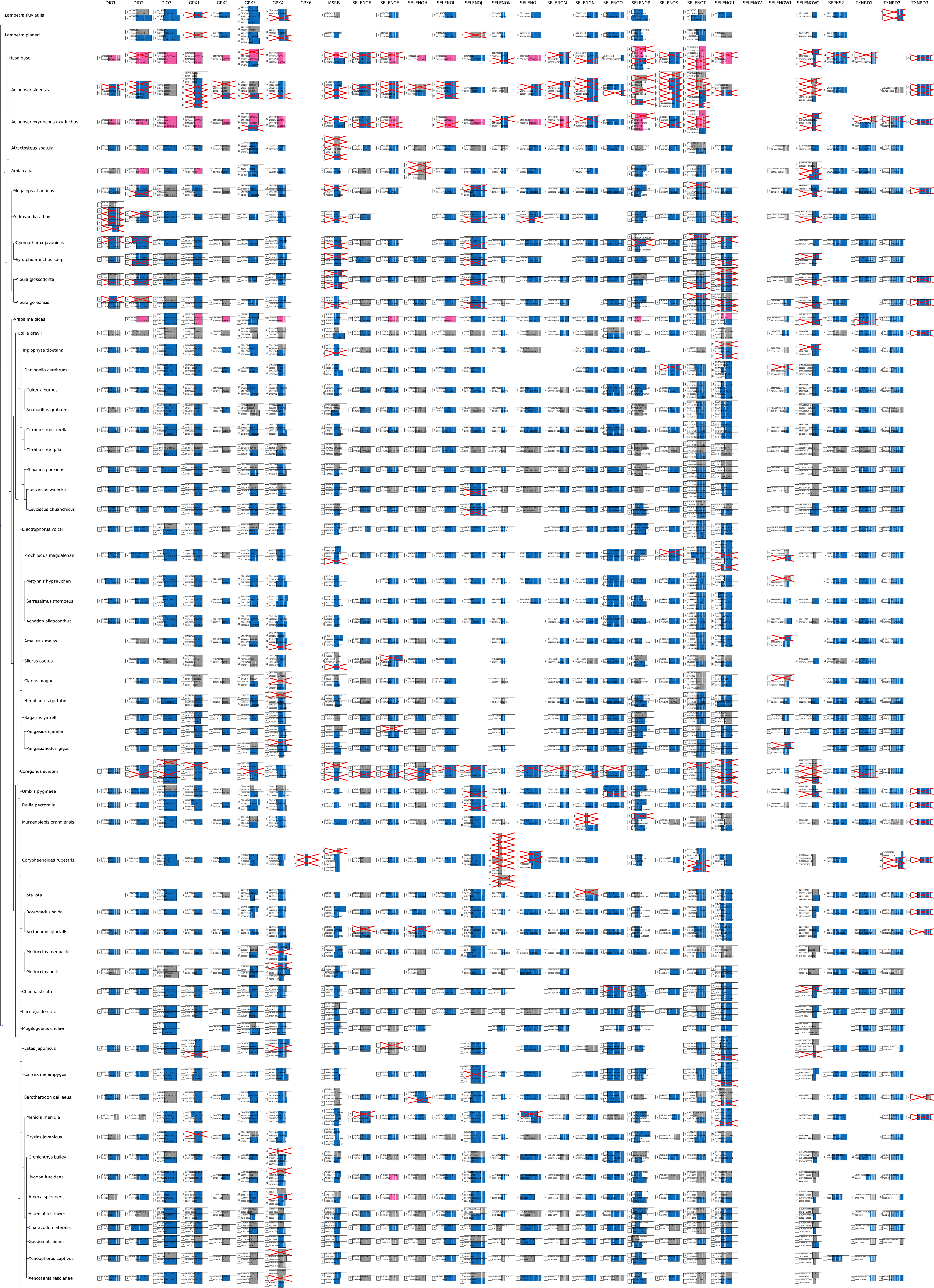
DIO1
DIO2
DIO3
GPX1
GPX2
GPX3
GPX4
GPX6
MSRB
SELENOE
SELENOF
SELENOH
SELENOI
SELENOJ
SELENOK
SELENOL
SELENOM
SELENON
SELENOO
SELENOP
SELENOS
SELENOT
SELENOU
SELENOV
SELENOW1
SELENOW2
SEPHS2
TXNRD1
TXNRD2
TXNRD3
Lampetra fluviatilis
Lampetra planeri
Huso huso
Acipenser sinensis
Acipenser oxyrinchus oxyrinchus
Atractosteus spatula
Amia calva
Megalops atlanticus
Aldrovandia affinis
Gymnothorax javanicus
Synaphobranchus kaupii
Albula glossodonta
Albula goreensis
Arapaima gigas
Coilia grayii
Triplophysa tibetana
Danionella cerebrum
Culter alburnus
Anabarilius grahami
Cirrhinus molitorella
Cirrhinus mrigala
Phoxinus phoxinus
Leuciscus waleckii
Leuciscus chuanchicus
Electrophorus voltai
Prochilodus magdalenae
Metynnis hypsauchen
Serrasalmus rhombeus
Acnodon oligacanthus
Ameiurus melas
Silurus asotus
Clarias magur
Hemibagrus guttatus
Bagarius yarrelli
Pangasius djambal
Pangasianodon gigas
Coregonus suidteri
Umbra pygmaea
Dallia pectoralis
Muraenolepis orangiensis
Coryphaenoides rupestris
Lota lota
Boreogadus saida
Arctogadus glacialis
Merluccius merluccius
Merluccius polli
Channa striata
Lucifuga dentata
Mugilogobius chulae
Lates japonicus
Caranx melampygus
Sarotherodon galilaeus
Menidia menidia
Oryzias javanicus
Crenichthys baileyi
Ilyodon furcidens
Ameca splendens
Ataeniobius toweri
Characodon lateralis
Goodea atripinnis
Xenoophorus captivus
Xenotaenia resolanae

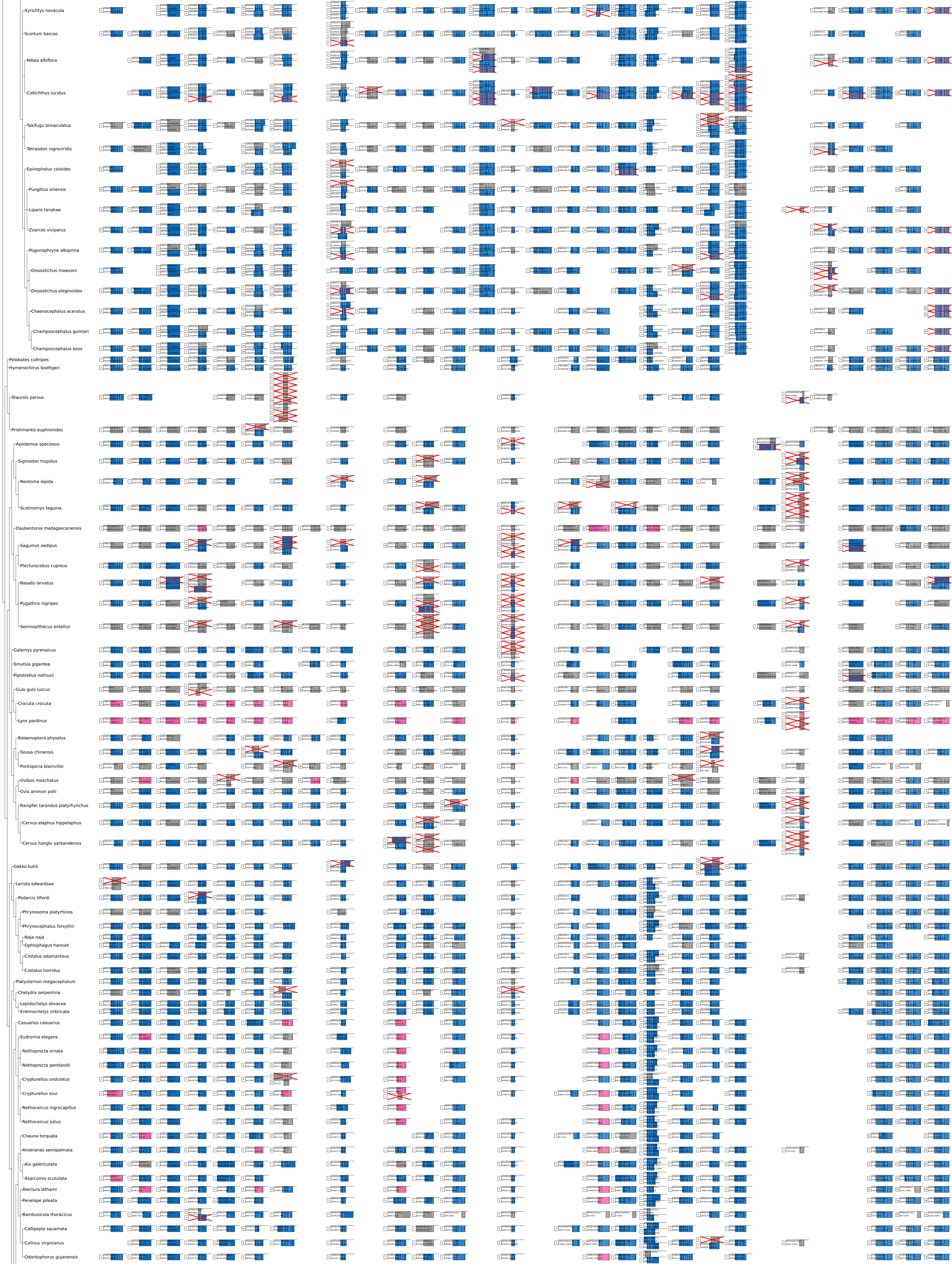

Xyrichtys novacula
Scortum barcoo
Nibea albiflora
Collichthys lucidus
Takifugu bimaculatus
Tetraodon nigroviridis
Epinephelus coioides
Pungitius sinensis
Liparis tanakae
Zoarces viviparus
Pogonophryne albipinna
Dissostichus mawsoni
Dissostichus eleginoides
Chaenocephalus aceratus
Champsocephalus gunnari
Champsocephalus esox
Pelobates cultripes
Hymenochirus boettgeri
Staurois parvus
Pristimantis euphronides
Apodemus speciosus
Sigmodon hispidus
Neotoma lepida
Scotinomys teguina
Daubentonia madagascariensis
Saguinus oedipus
Plecturocebus cupreus
Nasalis larvatus
Pygathrix nigripes
Semnopithecus entellus
Galemys pyrenaicus
Smutsia gigantea
Pipistrellus nathusii
Gulo gulo luscus
Crocuta crocuta
Lynx pardinus
Balaenoptera physalus
Sousa chinensis
Pontoporia blainvillei
Ovibos moschatus
Ovis ammon polii
Rangifer tarandus platyrhynchus
Cervus elaphus hippelaphus
Cervus hanglu yarkandensis
Gekko kuhli
Lerista edwardsae
Podarcis lilfordi
Phrynosoma platyrhinos
Phrynocephalus forsythii
Naja naja
Ophiophagus hannah
Crotalus adamanteus
Crotalus horridus
Platysternon megacephalum
Chelydra serpentina
Lepidochelys olivacea
Eretmochelys imbricata
Casuarius casuarius
Eudromia elegans
Nothoprocta ornata
Nothoprocta pentlandii
Crypturellus undulatus
Crypturellus soui
Nothocercus nigrocapillus
Nothocercus julius
Chauna torquata
Anseranas semipalmata
Aix galericulata
Asarcornis scutulata
Alectura lathami
Penelope pileata
Bambusicola thoracicus
Callipepla squamata
Colinus virginianus
Odontophorus gujanensis

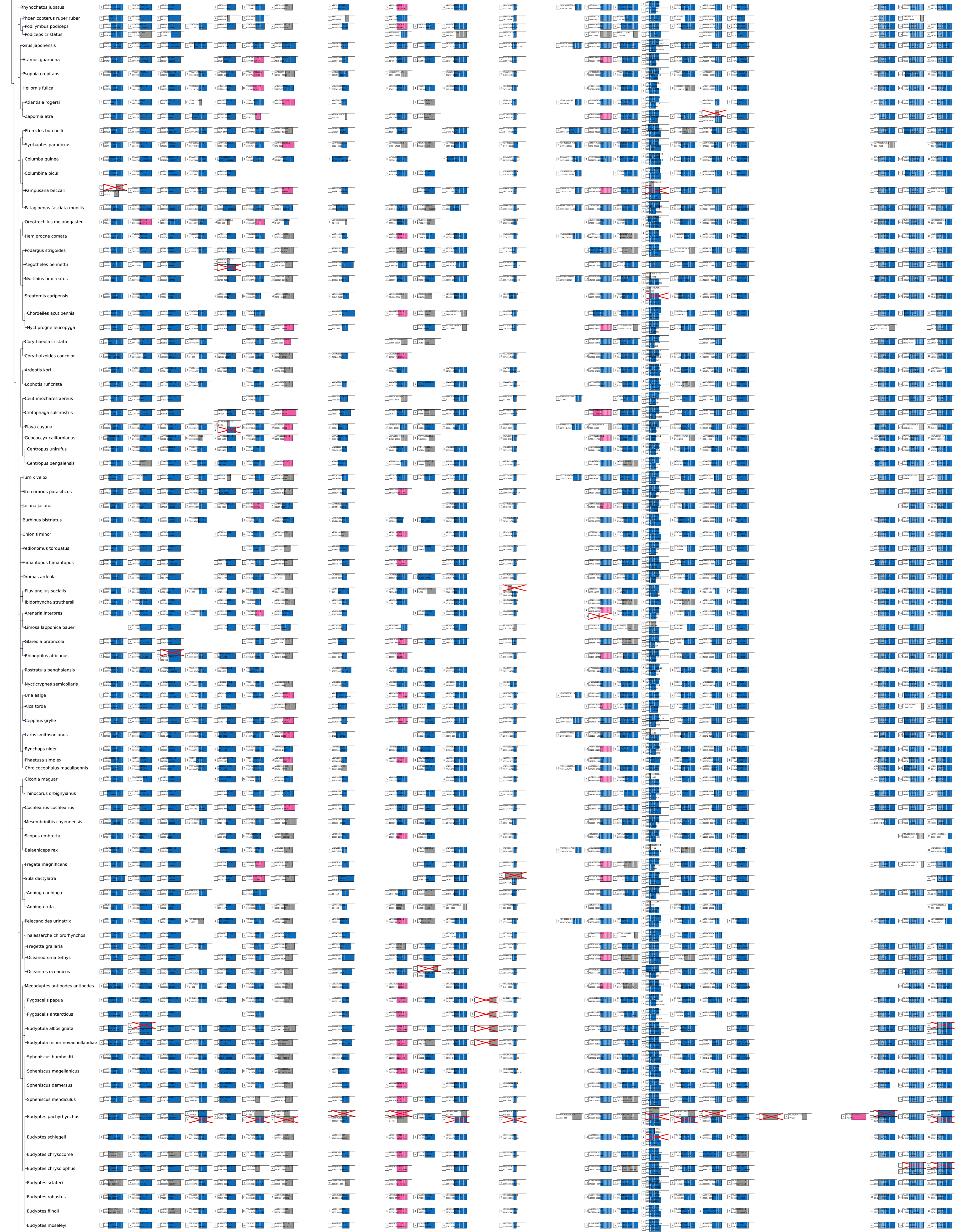
Rhynochetos jubatus
Phoenicopterus ruber ruber
Podilymbus podiceps
Podiceps cristatus
Grus japonensis
Aramus guarauna
Psophia crepitans
Heliornis fulica
Atlantisia rogersi
Zapornia atra
Pterocles burchelli
Syrrhaptes paradoxus
Columba guinea
Columbina picui
Pampusana beccarii
Patagioenas fasciata monilis
Oreotrochilus melanogaster
Hemiprocne comata
Podargus strigoides
Aegotheles bennettii
Nyctibius bracteatus
Steatornis caripensis
Chordeiles acutipennis
Nyctiprogne leucopyga
Corythaeola cristata
Corythaixoides concolor
Ardeotis kori
Lophotis ruficrista
Ceuthmochares aereus
Crotophaga sulcirostris
Piaya cayana
Geococcyx californianus
Centropus unirufus
Centropus bengalensis
Turnix velox
Stercorarius parasiticus
Jacana jacana
Burhinus bistriatus
Chionis minor
Pedionomus torquatus
Himantopus himantopus
Dromas ardeola
Pluvianellus socialis
Ibidorhyncha struthersii
Arenaria interpres
Limosa lapponica baueri
Glareola pratincola
Rhinoptilus africanus
Rostratula benghalensis
Nycticryphes semicollaris
Uria aalge
Alca torda
Cepphus grylle
Larus smithsonianus
Rynchops niger
Phaetusa simplex
Chroicocephalus maculipennis
Ciconia maguari
Thinocorus orbignyianus
Cochlearius cochlearius
Mesembrinibis cayennensis
Scopus umbretta
Balaeniceps rex
Fregata magnificens
Sula dactylatra
Anhinga anhinga
Anhinga rufa
Pelecanoides urinatrix
Thalassarche chlororhynchos
Fregetta grallaria
Oceanodroma tethys
Oceanites oceanicus
Megadyptes antipodes antipodes
Pygoscelis papua
Pygoscelis antarcticus
Eudyptula albosignata
Eudyptula minor novaehollandiae
Spheniscus humboldti
Spheniscus magellanicus
Spheniscus demersus
Spheniscus mendiculus
Eudyptes pachyrhynchus
Eudyptes schlegeli
Eudyptes chrysocome
Eudyptes chrysolophus
Eudyptes sclateri
Eudyptes robustus
Eudyptes filholi
Eudyptes moseleyi

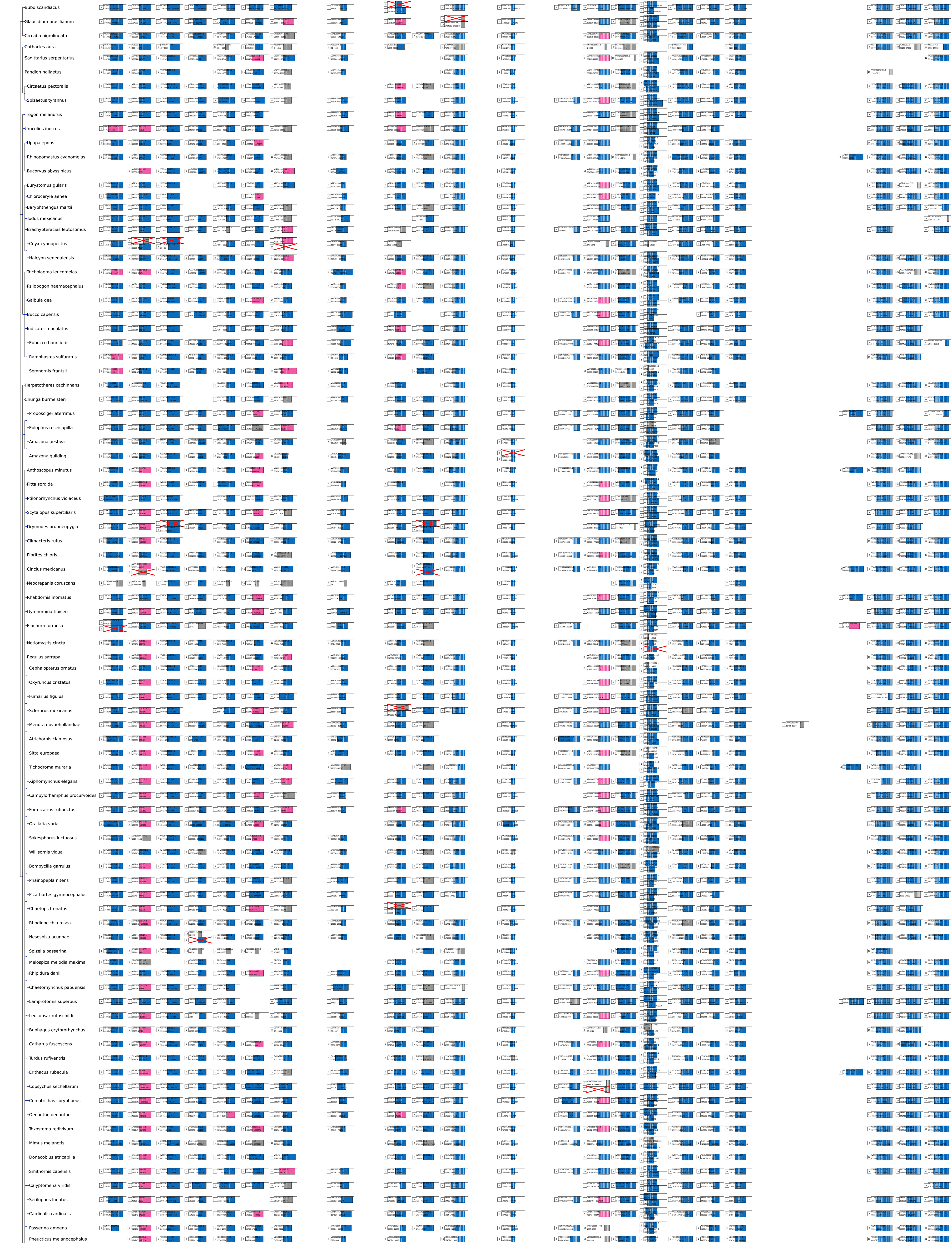
Bubo scandiacus
Glaucidium brasilianum
Ciccaba nigrolineata
Cathartes aura
Sagittarius serpentarius
Pandion haliaetus
Circaetus pectoralis
Spizaetus tyrannus
Trogon melanurus
Urocolius indicus
Upupa epops
Rhinopomastus cyanomelas
Bucorvus abyssinicus
Eurystomus gularis
Chloroceryle aenea
Baryphthengus martii
Todus mexicanus
Brachypteracias leptosomus
Ceyx cyanopectus
Halcyon senegalensis
Tricholaema leucomelas
Psilopogon haemacephalus
Galbula dea
Bucco capensis
Indicator maculatus
Eubucco bourcierii
Ramphastos sulfuratus
Semnornis frantzii
Herpetotheres cachinnans
Chunga burmeisteri
Probosciger aterrimus
Eolophus roseicapilla
Amazona aestiva
Amazona guildingii
Anthoscopus minutus
Pitta sordida
Ptilonorhynchus violaceus
Scytalopus superciliaris
Drymodes brunneopygia
Climacteris rufus
Piprites chloris
Cinclus mexicanus
Neodrepanis coruscans
Rhabdornis inornatus
Gymnorhina tibicen
Elachura formosa
Notiomystis cincta
Regulus satrapa
Cephalopterus ornatus
Oxyruncus cristatus
Furnarius figulus
Sclerurus mexicanus
Menura novaehollandiae
Atrichornis clamosus
Sitta europaea
Tichodroma muraria
Xiphorhynchus elegans
Campylorhamphus procurvoides
Formicarius rufipectus
Grallaria varia
Sakesphorus luctuosus
Willisornis vidua
Bombycilla garrulus
Phainopepla nitens
Picathartes gymnocephalus
Chaetops frenatus
Rhodinocichla rosea
Nesospiza acunhae
Spizella passerina
Melospiza melodia maxima
Rhipidura dahli
Chaetorhynchus papuensis
Lamprotornis superbus
Leucopsar rothschildi
Buphagus erythrorhynchus
Catharus fuscescens
Turdus rufiventris
Erithacus rubecula
Copsychus sechellarum
Cercotrichas coryphoeus
Oenanthe oenanthe
Toxostoma redivivum
Mimus melanotis
Donacobius atricapilla
Smithornis capensis
Calyptomena viridis
Serilophus lunatus
Cardinalis cardinalis
Passerina amoena
Pheucticus melanocephalus

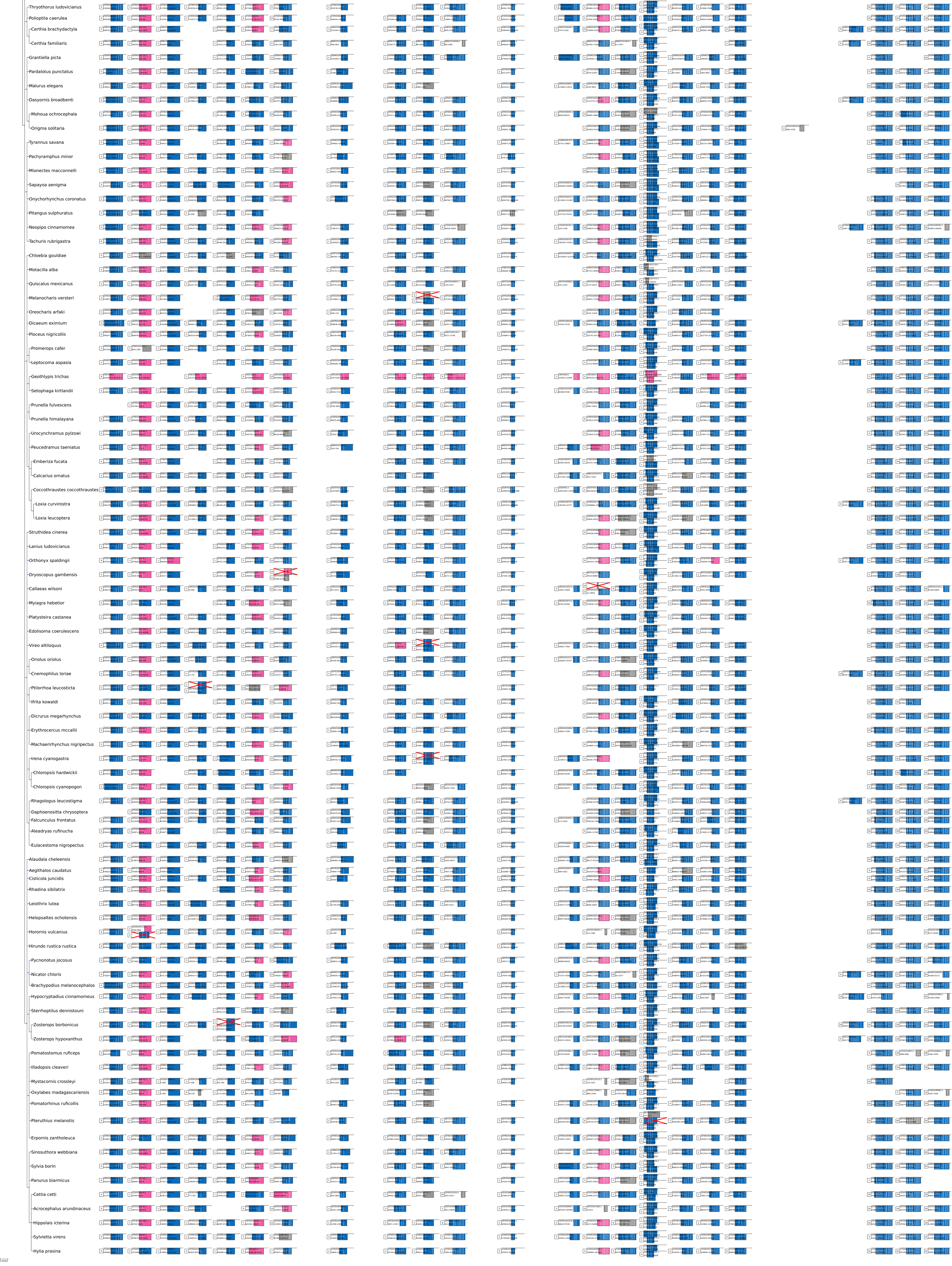

Thryothorus ludovicianus
Polioptila caerulea
Certhia brachydactyla
Certhia familiaris
Grantiella picta
Pardalotus punctatus
Malurus elegans
Dasyornis broadbenti
Mohoua ochrocephala
Origma solitaria
Tyrannus savana
Pachyramphus minor
Mionectes macconnelli
Sapayoa aenigma
Onychorhynchus coronatus
Pitangus sulphuratus
Neopipo cinnamomea
Tachuris rubrigastra
Chloebia gouldiae
Motacilla alba
Quiscalus mexicanus
Melanocharis versteri
Oreocharis arfaki
Dicaeum eximium
Ploceus nigricollis
Promerops cafer
Leptocoma aspasia
Geothlypis trichas
Setophaga kirtlandii
Prunella fulvescens
Prunella himalayana
Urocynchramus pylzowi
Peucedramus taeniatus
Emberiza fucata
Calcarius ornatus
Coccothraustes coccothraustes
Loxia curvirostra
Loxia leucoptera
Struthidea cinerea
Lanius ludovicianus
Orthonyx spaldingii
Dryoscopus gambensis
Callaeas wilsoni
Myiagra hebetior
Platysteira castanea
Edolisoma coerulescens
Vireo altiloquus
Oriolus oriolus
Cnemophilus loriae
Ptilorrhoa leucosticta
Ifrita kowaldi
Dicrurus megarhynchus
Erythrocercus mccallii
Machaerirhynchus nigripectus
Irena cyanogastra
Chloropsis hardwickii
Chloropsis cyanopogon
Rhagologus leucostigma
Daphoenositta chrysoptera
Falcunculus frontatus
Aleadryas rufinucha
Eulacestoma nigropectus
Alaudala cheleensis
Aegithalos caudatus
Cisticola juncidis
Rhadina sibilatrix
Leiothrix lutea
Helopsaltes ochotensis
Horornis vulcanius
Hirundo rustica rustica
Pycnonotus jocosus
Nicator chloris
Brachypodius melanocephalos
Hypocryptadius cinnamomeus
Sterrhoptilus dennistouni
Zosterops borbonicus
Zosterops hypoxanthus
Pomatostomus ruficeps
Illadopsis cleaveri
Mystacornis crossleyi
Oxylabes madagascariensis
Pomatorhinus ruficollis
Pteruthius melanotis
Erpornis zantholeuca
Sinosuthora webbiana
Sylvia borin
Panurus biarmicus
Cettia cetti
Acrocephalus arundinaceus
Hippolais icterina
Sylvietta virens
Hylia prasina
